# Integrative multi-omics analysis unravels the host response landscape and reveals a serum protein panel for early prognosis prediction for ARDS

**DOI:** 10.1186/s13054-024-05000-3

**Published:** 2024-07-02

**Authors:** Mengna Lin, Feixiang Xu, Jian Sun, Jianfeng Song, Yao Shen, Su Lu, Hailin Ding, Lulu Lan, Chen Chen, Wen Ma, Xueling Wu, Zhenju Song, Weibing Wang

**Affiliations:** 1 Shanghai Institute of Infectious Disease and Biosecurity, School of Public Health, Fudan University, Shanghai, China; 2 Department of Emergency Medicine, Zhongshan Hospital, Fudan University, Shanghai, China; 3 Department of Emergency Medicine, Minhang Hospital, Fudan University, Shanghai, China; 4 Department of Respiratory Medicine, Pudong Hospital, Fudan University, Shanghai, China; 5 School of Public Health, Fudan University, Shanghai, China; 6 Department of Respiratory Medicine, Renji Hospital, Shanghai Jiaotong University School of Medicine, Shanghai, China; 7 Institute of Emergency Rescue and Critical Care, Fudan University, Shanghai, China

**Keywords:** Proteomics, Metabolomics, Multi-omics, ARDS, Machine learning, Prognosis model

## Abstract

**Background:**

The multidimensional biological mechanisms underpinning acute respiratory distress syndrome (ARDS) continue to be elucidated, and early biomarkers for predicting ARDS prognosis are yet to be identified.

**Methods:**

We conducted a multicenter observational study, profiling the 4D-DIA proteomics and global metabolomics of serum samples collected from patients at the initial stage of ARDS, alongside samples from both disease control and healthy control groups. We identified 28-day prognosis biomarkers of ARDS in the discovery cohort using the LASSO method, fold change analysis, and the Boruta algorithm. The candidate biomarkers were validated through parallel reaction monitoring (PRM) targeted mass spectrometry in an external validation cohort. Machine learning models were applied to explore the biomarkers of ARDS prognosis.

**Results:**

In the discovery cohort, comprising 130 adult ARDS patients (mean age 72.5, 74.6% male), 33 disease controls, and 33 healthy controls, distinct proteomic and metabolic signatures were identified to differentiate ARDS from both control groups. Pathway analysis highlighted the upregulated sphingolipid signaling pathway as a key contributor to the pathological mechanisms underlying ARDS. MAP2K1 emerged as the hub protein, facilitating interactions with various biological functions within this pathway. Additionally, the metabolite sphingosine 1-phosphate (S1P) was closely associated with ARDS and its prognosis. Our research further highlights essential pathways contributing to the deceased ARDS, such as the downregulation of hematopoietic cell lineage and calcium signaling pathways, contrasted with the upregulation of the unfolded protein response and glycolysis. In particular, GAPDH and ENO1, critical enzymes in glycolysis, showed the highest interaction degree in the protein–protein interaction network of ARDS. In the discovery cohort, a panel of 36 proteins was identified as candidate biomarkers, with 8 proteins (VCAM1, LDHB, MSN, FLG2, TAGLN2, LMNA, MBL2, and LBP) demonstrating significant consistency in an independent validation cohort of 183 patients (mean age 72.6 years, 73.2% male), confirmed by PRM assay. The protein-based model exhibited superior predictive accuracy compared to the clinical model in both the discovery cohort (AUC: 0.893 *vs.* 0.784; Delong test, *P* < 0.001) and the validation cohort (AUC: 0.802 *vs.* 0.738; Delong test, *P*  = 0.008).

**Interpretation:**

Our multi-omics study demonstrated the potential biological mechanism and therapy targets in ARDS. This study unveiled several novel predictive biomarkers and established a validated prediction model for the poor prognosis of ARDS, offering valuable insights into the prognosis of individuals with ARDS.

**Supplementary Information:**

The online version contains supplementary material available at 10.1186/s13054-024-05000-3.

## Introduction

ARDS represents a prevalent manifestation of critical illness, arising from severe infections, major injuries, or the inhalation of harmful irritants [1]. Research indicates that ARDS affects approximately 10% of patients in intensive care units (ICUs), with mortality reaching up to 46% and even 70% during the coronavirus disease 2019 (COVID-19) pandemic [2]. Consequently, there is a need to seek effective treatments and precise prognostic methods to enhance ARDS patient outcomes, a need more intensified by the COVID-19 crisis. However, predicting ARDS prognosis in the early days after ARDS diagnosis is challenging due to the high variability in the underlying mechanisms of ARDS. The systemic response alterations observed in ARDS can be attributed to various factors, including pathogen and injury exposure, genetic susceptibility, and immune responses [3]. These factors may influence protein activity and the downstream metabolites that drive disease progression [4–6]. Therefore, it is crucial to investigate the potential association between host-derived proteins and metabolites circulating in the bloodstream with the pathogenesis and advancement of ARDS.

The integration of multi-omics approaches, particularly the combination of proteomics and metabolomics, elucidates the interactions across different biological system layers, as demonstrated in studies on cardiomyopathy [7], non‑small cell lung cancer [8], hepatitis C infection [9], etc. Previous studies have utilized serum proteins or metabolites to investigate infectious diseases. For example, one study identified proteins capable of accurately distinguishing and predicting COVID-19 outcomes [5]. Jacob *et al.* used proteomics and metabolomics to identify early predictive and pathogenic signatures of *Staphylococcus aureus* bacteremia [10]. Yi Wang and his colleagues investigated new diagnosis biomarkers and potential mechanisms in pediatric severe community-acquired pneumonia using proteomics combined with metabolomics [11]. However, the interplay between proteomic and metabolic profiles in the progression of adult ARDS and their collective role from a holistic perspective remains underexplored. Moreover, blood biomarkers have gained significant interest in ARDS investigations in recent years [12], showing promise in enhancing diagnosis, prognostication, and management strategies for ARDS, either independently or in conjunction with physiological parameters.

The primary objective of this study is to utilize proteomics and metabolomics to investigate the host response in ARDS within a discovery cohort comprising ARDS onset, disease, and healthy controls. This approach enabled a comprehensive profiling of biological characteristics and potential mechanisms associated with ARDS. The second objective is to screen the candidate biomarkers of ARDS prognosis based on proteomics and machine learning methods. Then, we employed the Parallel Reaction Monitoring (PRM) method to validate the prognosis biomarkers at ARDS onset in an independent cohort. The multi-omic analysis generated in this study provided a global overview of the molecular changes, which may provide useful insight into the therapy and prognosis of ARDS.

## Material and methods

### Ethical approval

The study involving human participants was reviewed and approved by the Ethical Committee of Zhongshan Hospital, Fudan University, China (Ethical approval number: B2023-029R), Renji Hospital, Shanghai Jiaotong University School of Medicine, China (No. LY2023-096-B), Minhang Hospital, Fudan University, China (No. 2022-041-01K), and Pudong Hospital, Fudan University, China (No. WZ-22). Written informed consent to participate in this study was provided by the participants or their relatives.

### Collections and preparation of clinical specimen

#### Discovery cohort

Single-center observational study. ARDS patients were recruited from the ICU of Zhongshan Hospital between December 2022 and September 2023. The ARDS cohort included patients aged over 18 years who were admitted to the ICU and clinically diagnosed with ARDS according to the Berlin definition [13]. Serum samples were collected within 48 hours after diagnosis. Patients whose diagnosis of ARDS was agreed upon by at least two out of three clinical experts were included. A comprehensive analysis of clinical data was performed, utilizing patient medical records to gather information on routine blood tests, liver function tests (including alanine aminotransferase [ALT] and aspartate aminotransferase [AST]), total and conjugated bilirubin (STB and CB), renal function indicators (blood urea nitrogen [BUN] and creatinine [Cr]), albumin [Alb], C-reactive protein [CRP)], procalcitonin [PCT], and coagulation profiles (fibrinogen [Fg], prothrombin time [PT], activated partial thromboplastin time [APTT], and D-dimer). The Sepsis-related Organ Failure Assessment (SOFA) score and PaO_2_/FiO_2_ ratio (P/F ratio) were evaluated concurrently. Comorbidities were also collected. For comparative analysis, disease controls (DC) and healthy controls (HC) were recruited and matched with the ARDS cohort based on age and sex. The inclusion criteria for the DC group were as follows: patients aged > 18 years, patients admitted to the ICU and had risk factors (sepsis, pneumonia, etc.) of ARDS, serum samples will be collected within the first 48 hours of ICU admission. We finally chose who did not progress to ARDS during the hospital stay. Patients who transferred to the other hospital were excluded. The HC group was derived from individuals registered at the Health Check Center of Zhongshan Hospital. HC controls were selected based on the absence of acute diseases such as infection and the lack of significant abnormalities on chest radiography. The control groups were matched for age and sex with the ARDS patients. The details for inclusion and exclusion for ARDS, DC, and HC groups are in the Supporting Information (Fig. S1).

#### Validation cohort

Multi-center validation. Validation samples were from the ICUs of the other three hospitals. Serum samples from patients diagnosed with ARDS were collected within 48 hours after the onset of ARDS. All venous blood was collected from participants and processed within 8 hours to isolate serum. The serum was separated by centrifugation at 300 × g for 10 min and stored at − 80 °C until testing.

#### Proteomics sequencing and data preprocessing

Proteins were enriched from serum using magnetic nanomaterials [14]. The mass spectrometer was operated in data-independent acquisition (DIA) mode. The DIA raw data were processed using Spectronaut Pulsar 17.5 software (Biognosys) against the Uniprot-Homo sapiens-9606-2023.2.1.fasta database. The details of proteomics sequencing and data preprocessing were in Supporting Information.

#### Metabolomics sequencing and data preprocessing

A Dionex Ultimate 3000 RS UHPLC fitted with Q-Exactive plus quadrupole-Orbitrap mass spectrometer equipped with heated electrospray ionization (ESI) source (Thermo Fisher Scientific, Waltham, MA, USA) was used to analyze the metabolic profiling in both ESI positive and ESI negative ion modes. The original liquid chromatography coupled with high-resolution mass spectrometry (LC-MS) data was processed by software Progenesis QI V2.3 (Nonlinear, Dynamics, Newcastle, UK) for baseline filtering, peak identification, integral, retention time correction, peak alignment, and normalization. The main parameters of 5 ppm precursor tolerance, 10 ppm product tolerance, and 5% production threshold were applied. Compound identification was based on the precise mass-to-charge ratio (M/z), secondary fragments, and isotopic distribution using The Human Metabolome Database (HMDB), Lipidmaps (V2.3), Metlin, EMDB, PMDB, and self-built databases to do qualitative analysis. The details of data preprocessing were in Supporting Information.

#### Validation of candidate biomarkers using PRM assay

To evaluate the candidate biomarkers of prognosis, a unique PRM assay was generated, incorporating as many candidate proteins as possible. For the proteome profiling samples, the peptide was examined utilizing a Q Exactive HF-X Hybrid Quadrupole-Orbitrap Mass Spectrometer (Thermo Fisher Scientific), integrated with a state-of-the-art high-performance liquid chromatography system (EASY nLC 1200, Thermo Fisher Scientific). The experimental details of the PRM assay were in Supporting Information.

### Bioinformatic analysis

#### Data quality control and pre-processing of proteomics

The proteomics data analysis encompassed the following key procedures mentioned in former studies [15, 16]. Step 1: Screen proteins with unique peptides ≥ 1 for further analysis. Step 2: Proteins with more than 50% null values in three groups were excluded. Step 3: For proteins whose effective value is ≥ 50% in one group, the empty values were filled with the mean value of the group. The remaining missing values were filled using half of the sample’s minimum value. Step 4: high-quality proteins were retained by Log2 transformation and z-normalization for subsequent data analysis. Specifically, 2669 proteins were collected for downstream statistical and bioinformatics analysis.

#### Screening of differentially abundant proteins (DAP), differentially abundant metabolites (DAM), and enrichment analysis

Protein abundance changes in different sample groups were conducted through principal component analysis (PCA). For circulating proteomic data from the discovery cohort, the fold change (FC) value was derived from the ratio of ARDS to non-ARDS cases. Statistically significant differentially abundant proteins (DAPs) and differentially abundant metabolites (DAMs) were identified based on the criteria of FC ≥ 2 or FC ≤ − 2, and *P* < 0.05. *P*-values were adjusted for false discovery rate (FDR) using Benjamini and Hochberg. We performed Kyoto Encyclopedia of Genes and Genomes (KEGG) enrichment to investigate further potential pathological and biological mechanisms associated with ARDS. The functional enrichment analysis of KEGG was performed with the ClusterProfiler package in *R*. KEGG pathway database (https://www.kegg.jp/kegg/pathway.html) was applied for metabolites pathway enrichment analysis.

Utilizing the results from KEGG pathways, we identified key interactions between enriched proteins and metabolites in ARDS. These elements were systematically linked to their corresponding pathways to elucidate the molecular mechanisms potentially influencing ARDS development. This linkage is visually represented through network diagrams, created by Cytoscape [17].

#### Protein-protein interaction network analysis

The protein-protein interactions (PPIs) were obtained from the STRING database [18]. Differentially abundant proteins (*P*-value < 0.05) were mapped to PPIs to generate the DAP PPI network in ARDS. The Cytoscape software [17] was used to visualize the network. The Cytoscape plugin cytoHubba [19] was utilized to calculate the degree in the PPI network.

#### Gene set enrichment analysis (GSEA) analysis

For the GSEA enrichment analysis, we utilized the Molecular Signatures Database (MSigDB), specifically focusing on the KEGG gene set. We designated an FDR threshold of 0.05 as the boundary for statistical significance. The process for computing the Normalized Enrichment Score (NES) within the GSEA framework entails the prioritization of proteins based on their statistical relevance, ranging from the most to the least significant, succeeded by the examination of the distribution pattern of the proteins associated with each gene set throughout the prioritized list. The integrated abundance of proteins was then calculated by utilizing the ClusterProfiler package of *R* [20].

#### Constructing a prognostic model based on the early serum proteome

We constructed three classification models to predict adverse outcomes of ARDS: first, screened candidate proteins were used to construct a protein-based model. Least absolute shrinkage and selection operator (LASSO) and Boruta methods were used to screen the candidate proteins in the discovery cohort, then combined with DAPs, candidate biomarkers with unique peptides ≥ 3 were proposed for further targeted proteomics analysis. Second, to compare the protein model with current clinical practice, a clinical risk model was constructed and optimized. The clinical parameters were selected by the LASSO method among the lab tests, SOFA, P/F ratio, and comorbidity. A third combined model was formed by stacking the clinical prognostic parameters with the protein parameters. Five machine learning methods, Naïve Bayes (Bayes), Random forest (RF), Generalized Linear Model (Glm), Supporting Vector machine (SVM), and Gradient Boosting Machine (GBM) were used in the three classification models. Discrimination performance was assessed using the receiver operating characteristic (ROC) curve with an area under the curve (AUC), sensitivity, and specificity. The DeLong test was strategically utilized to statistically compare the AUCs across different models, providing a robust assessment of their discriminative capabilities. To prevent overfitting, ‘10-fold cross-validation’ was employed to assess the performance of machine learning methods.

## Statistical analysis

Mann–Whitney *U* tests or Kruskal–Wallis tests were used for comparisons of continuous variables, whereas *Chi-square* tests were used for categorical variables. Correlation analysis was assessed by *Spearman* correlation. A *P* value of < 0.05 (two-tailed) was considered statistically significant Benjamini–Hochberg correction for multiple testing was applied as appropriate.

## Results

### Participants characteristics

**Fig. 1:**
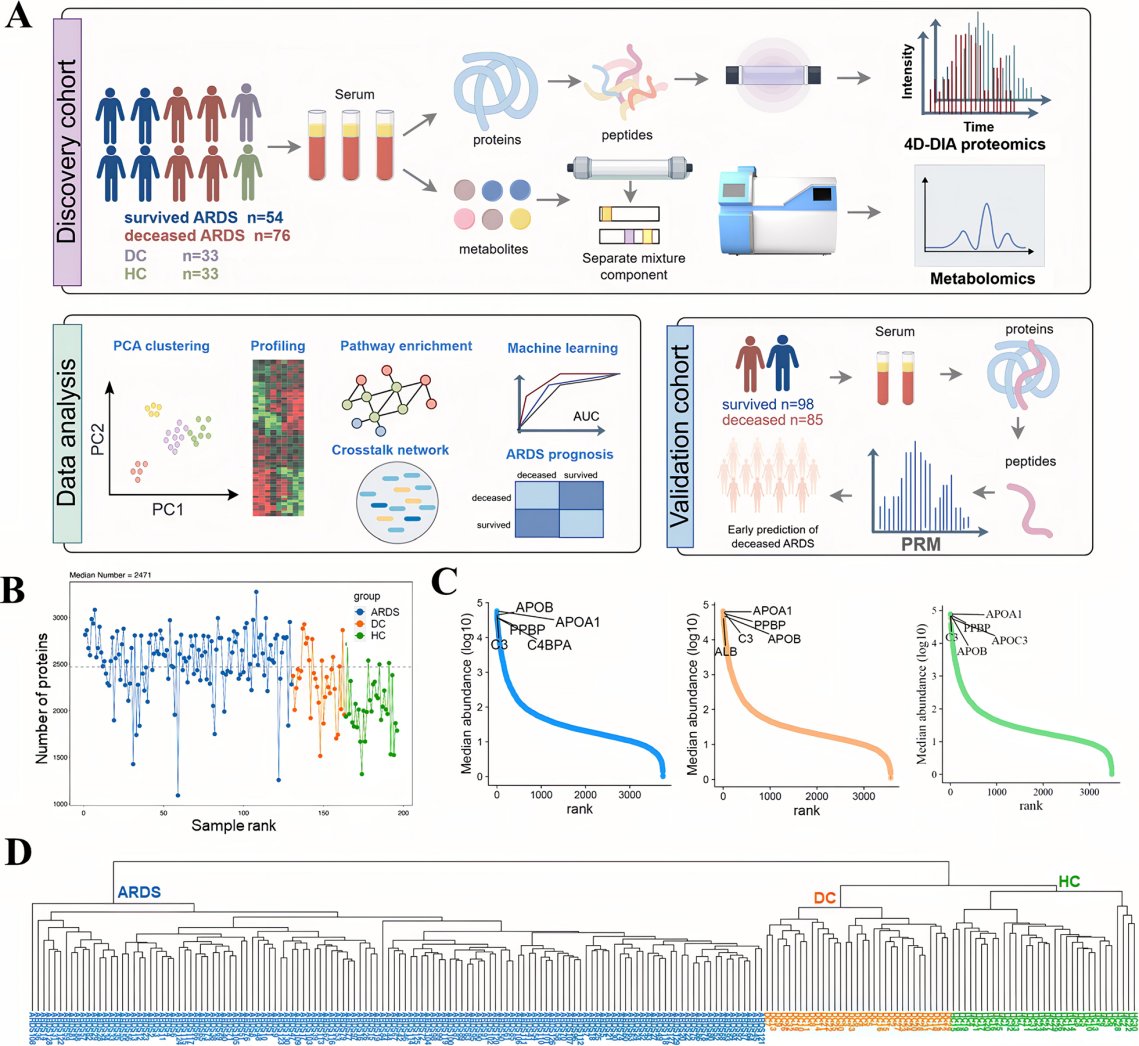
Study design overview and summary of the serum proteomic analysis of ARDS, disease control, and health control. **A** Overview of the serum proteomic and metabolic workflow, including cohort construction (survived ARDS: N = 54, deceased ARDS: N = 76, disease control: N = 33, and healthy control: N = 33), serum validation cohort (survived ARDS: N = 98 and deceased ARDS: N = 85) and data analysis (proteomic and metabolic data). **B** A number of proteins were identified and quantified in three groups. **C** The top five most abundant proteins are labeled. **D** The clustering tree of each sample

The characteristics of the validation and discovery cohorts are in Table 1. There was no significant difference in the ARDS etiologies between the discovery and validation cohorts. For the discovery cohort, the demographic and clinical characteristics of 130 ARDS cases, including 54 survived ARDS and 76 deceased ARDS are presented in Table 2. At baseline, the mean age of the ARDS group was 72.5 (SD, 11.4) years, and 74.6% were male. After the diagnosis of ARDS, non-survivors showed decreased P/F ratio, PLT, and Alb levels, alongside increased SOFA scores, BUN, and AST, compared to the survivors. Gender distribution and age were matched in DC and HC with ARDS (Table 1). We acquired serum proteome profiles of all participants (*n* = 196) using a DIA strategy and global metabolome by the LC-MS method. An overview of proteomics and metabolomics workflow is showed in Fig. 1A. The independent prospective validation cohort incorporating 183 early ARDS patients with 85 deceased and 98 survived (Fig. 1A and Table S1). The characteristics of the validation and discovery cohorts were nearly consistent, except for Alb and Cr (Table 2 and Table S1).

**Table 1:** Baseline characteristics of the participants in the discovery and validation cohorts

	ARDS in the discovery cohort (N = 130)	ARDS in the validation cohort (N = 183)	Disease control (N = 33)	Healthy control (N = 33)	*P1*	*P2*
Age (mean, SD)	72.5 (11.4)	72.6 (14.3)	72.1 (11.6)	72.0 (8.2)	0.889	0.883
Male (n, %)	97 (74.6)	134 (73.2)	25 (75.8)	25 (75.8)	0.783	0.774
SOFA	6 [4–9]	4 [3–7]	3 [2, 3]	–	0.001**	< 0.001***
P/F ratio	109 [81–151]	150.0 [90.0–239.0]	265 [203–354]	–	0.001**	< 0.001***
RBC (*10^12^/L)	3.66 [2.90–4.11]	3.91 [3.28–4.28]	4.10 [3.77–4.50]	–	0.099	0.367
Hb (g/L)	111.0 [90.0–128.0]	115.0 [96.0–131.0]	127.5 [113.3–137.3]	–	0.167	0.657
WBC (*10^9^/L)	10.35 [7.65–14.30]	10.51 [7.36–14.50]	7.2 [5.4–11.2]	–	0.761	0.676
NEU (*10^9^/L)	9.30 [6.60–12.45]	9.0 [6.2–13.0]	5.3 [3.7–9.7]	–	0.968	0.352
LYM (*10^9^/L)	0.4 [0.2–0.6]	0.6 [0.4–1.0]	0.8 [0.5–1.1]	–	0.001**	0.234
PLT (*10^9^/L)	153.5 [82.5–229.3]	155.0 [110.0–222.0]	191.0 [151.5–254.0]	–	0.545	0.005**
ALT (U/L)	27.0 [18.0–45.3]	30.0 [18.0–53.0]	30.0 [22.0–41.0]	–	0.282	0.561
AST (U/L)	29.0 [20.0–47.0]	34.0 [21.0–53.0]	28.0 [21.0–50.8]	–	0.158	0.041*
STB (µmol/L)	11.4 [7.6–19.2]	11.10 [8.60–20.60]	9.9 [6.7–11.7]	–	0.218	0.931
CB (µmol/L)	4.9 [3.1–8.4]	4.10 [3.00–6.70]	3.9 [3.0–5.2]	–	0.479	0.938
BUN (mmol/L)	12.15 [9.18–20.98]	9.30 [6.10–15.00]	6.9 [5.5–9.0]	–	0.001**	0.049*
Cr (µmol/L)	90.0 [64.8–128.8]	79.0 [63.0–113.0]	77.5 [62.5–97.8]	–	0.131	0.097
Alb (g/L)	31.0 [28.0–34.0]	32.0 [29.0–35.0]	34.0 [32.0–37.0]	–	0.058	0.006**
CRP (mg/L)	60.3 [19.6–90.0]	64.0 [23.4–145.1]	23.9 [4.4–62.4]	–	0.020*	0.109
PCT (ng/mL)	0.29 [0.11–0.61]	0.26 [0.11–1.22]	0.07 [0.03–0.19]	–	0.177	0.568
Fg (mg/dL)	419.5 [278.3–540.3]	448.0 [212.0–526.0]	469.0 [269.0–537.0]	–	0.655	0.921
PT (second)	13.3 [12.5–14.7]	13.5 [12.6–15.1]	12.6 [12.0–13.3]	–	0.456	0.079
APTT (second)	29.3 [26.4–34.3]	29.2 [26.8–35.5]	27.6 [25.9–30.9]	–	0.108	0.944
D-dimer (mg/L)	4.22 [1.75–10.7]	2.86 [1.28–8.06]	1.13 [0.57–3.31]	–	0.079	0.158
Hypertension (n, %)	87 (66.9)	90 (49.2)	23 (69.7)	–	0.002**	0.607
Coronary heart disease (n, %)	30 (23.1)	36 (19.7)	7 (21.2)	–	0.468	1.000
Diabetes (n, %)	50 (38.5)	57 (31.1)	14 (42.4)	–	0.180	0.054
Cerebrovascular disease (n, %)	23 (17.7)	30 (16.4)	2 (6.1)	–	0.763	0.659
Immunosuppression (n, %)	13 (10.0)	20 (10.9)	1 (3.0)	–	0.792	0.953
Vasopressor use at enrolment (n, %)	24 (18.5)	34 (18.6)	3 (9.1)	–	1.000	0.303
Neuromuscular blockade use at enrolment (n, %)	39 (30.0)	50 (27.3)	0 (0)	–	0.696	< 0.001***
Mechanical ventilation (n, %)	118 (90.8)	165 (90.2)	0 (0)	–	1.000	< 0.001***
Prone positioning at enrolment (n, %)	70 (53.8)	96 (52.5)	0 (0)	–	0.899	< 0.001***
Risk factors (n, %)					0.962	0.882
Pneumonia	96 (73.8)	137 (74.9)	26 (75.8)	–		
Sepsis	26 (20.0)	36 (19.7)	6 (18.2)	–		
Other	8 (6.2)	10 (5.5)	1 (3.0)	–		
Source of infection (n, %)					0.928	0.786
Thorax	96 (73.8)	137 (74.9)	26 (75.8)	–		
Abdomen	24 (18.5)	34 (18.6)	6 (18.2)	–		
Other	10 (7.7)	12 (6.5)	1 (3.0)	–		

*P1*: Comparison between ARDS patients in the discovery cohort and validation cohort; *P2*: Comparison between ARDS patients in the discovery cohort and Disease control

*P < 0.05, **P < 0.01, ***P < 0.001

**Table 2:** Baseline characteristics of the discovery cohort

	Survived ARDS (n = 54)	Deceased ARDS (n = 76)	*P*
Age (mean, SD)	72.2 (11.8)	73.7 (11.2)	0.883
Male (n, %)	41 (75.9)	56 (73.7)	0.774
SOFA	5 [4–7]	6 [5–9]	< 0.001***
P/F ratio	122 [95–200]	98 [73–138]	< 0.001***
RBC (*10^12^/L)	3.55 [2.88–4.00]	3.71 [2.95–4.16]	0.367
Hb (g/L)	108.5 [85.8–128.0]	111.5 [91.3–127.8]	0.657
WBC (*10^9^/L)	9.83 [7.56–12.32]	10.41 [7.66–15.04]	0.676
NEU (*10^9^/L)	8.80 [6.38–10.65]	9.55 [6.70–13.28]	0.352
LYM (*10^9^/L)	0.4 [0.3–0.8]	0.4 [0.2–0.6]	0.234
PLT (*10^9^/L)	189.5 [126.8–249.5]	129.0 [68.0–205.0]	0.005**
ALT (U/L)	29.5 [19.8–54.8]	25.5 [17.0–44.0]	0.561
AST (U/L)	27.0 [20.0–34.0]	34.0 [21.0–54.0]	0.041*
STB (µmol/L)	10.8 [7.7–17.6]	12.3 [7.4–20.1]	0.931
CB (µmol/L)	4.5 [3.2–6.5]	5.5 [3.1–8.6]	0.938
BUN (mmol/L)	11.55 [9.45–15.85]	12.8 [8.6–24.38]	0.049*
Cr (µmol/L)	81.0 [63.5–111.8]	96.0 [67.5–169.8]	0.097
Alb (g/L)	32.0 [29.0–35.3]	30.0 [28.0–32.0]	0.006**
CRP (mg/L)	55.1 [8.6–68.3]	68.9 [26.3–98.3]	0.109
PCT (ng/mL)	0.23 [0.08–0.76]	0.30 [0.11–0.56]	0.568
Fg (mg/dL)	410.5 [249.8–560.3]	427.0 [301.0–538.0]	0.921
PT (second)	13.1 [12.2–14.0]	13.5 [12.7–15.1]	0.079
APTT (second)	29.4 [25.6–34.2]	29.3 [27.0–34.3]	0.944
D-dimer (mg/L)	3.27 [1.42–7.13]	4.41 [2.02–11.50]	0.158
Hypertension (n, %)	38 (70.4)	49 (64.5)	0.607
Coronary heart disease (n, %)	12 (22.2)	18 (23.7)	1.000
Diabetes (n, %)	15 (27.8)	35 (46.1)	0.054
Cerebrovascular disease (n, %)	11 (20.4)	12 (15.8)	0.659
Immunosuppression (n, %)	6 (11.1)	7 (9.2)	0.953
Vasopressor use at enrolment (n, %)	8 (14.8)	16 (21.1)	0.500
Neuromuscular blockade use at enrolment (n, %)	14 (25.9)	25 (32.9)	0.509
Mechanical ventilation (n, %)	48 (88.9)	70 (92.1)	0.553
Prone positioning at enrolment (n, %)	30 (55.6)	40 (52.6)	0.880
ARDS risk factors (n, %)			0.397
Pneumonia	42 (77.8)	54 (71.0)	
Sepsis	8 (14.8)	18 (23.7)	
Other	4 (7.4)	4 (5.3)	
Source of infection (n, %)			0.369
Thorax	42 (77.8)	54 (71.0)	
Abdomen	7 (13.0)	17 (22.4)	
Other	5 (9.2)	5 (6.6)	

*P < 0.05, **P < 0.01, ***P < 0.001

### Altered serum proteomic profiling in the ARDS group and the biological pathways

**Fig. 2:**
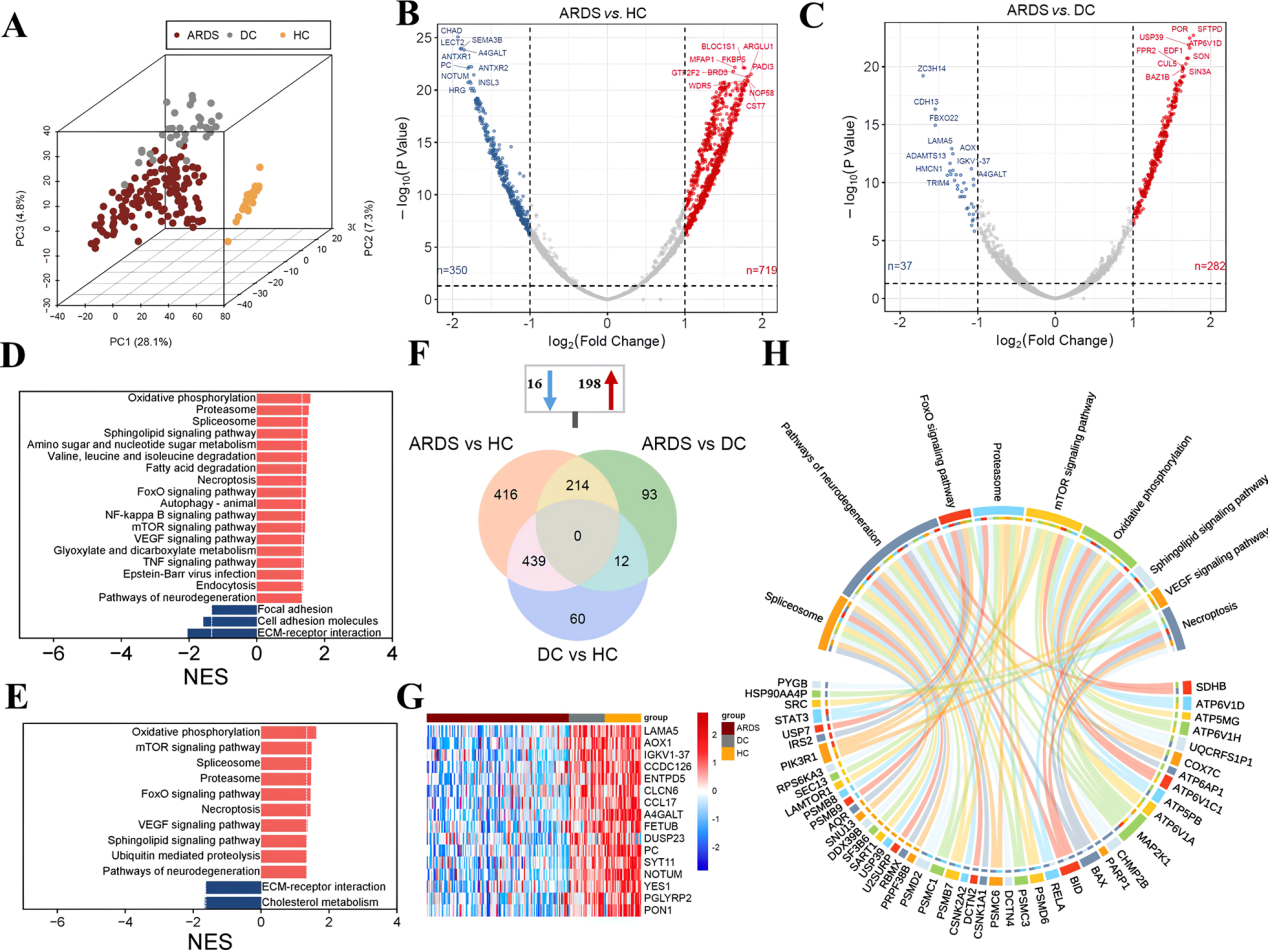
Differentially abundant proteins and functional alterations in ARDS. **A** PCA discriminates ARDS patients from non-ARDS subjects. The % value indicates the explained variance. Volcano plot displaying differentially abundant proteins between ARDS *vs.* HC **B** and ARDS *vs.* DC **C**. Each dot represents a protein, with red dots for proteins significantly upregulated in ARDS and blue dots for proteins significantly downregulated in ARDS. *P* values were calculated using the *R* package ‘limma’ and adjusted using the Benjamini–Hochberg method. Two-sided *P* values were calculated. Fold change ≥ 2.0 and FDR-adjusted *P* < 0.05 is considered statistically significant. **D–E** KEGG pathway enrichments of ARDS compared with HC and DC groups, respectively. **F** A Venn diagram elucidates the distribution of DAP across the ARDS, DC, and HC groups. **G** The heatmap showed the 16 overlapped down-regulated proteins in ARDS. **H** A chordal graph maps the interaction between 9 common pathways and their associated proteins

After filtering the low abundant proteins, 2669 high-quality proteins were collected for the data analysis (Additional file 1). The median protein number for 196 samples was 2471 (Fig. 1B–C). The clustering tree indicated the appropriate pre-processing method in this study (Fig. 1D). Our preliminary analysis employed PCA method to explore the clustering patterns among the groups (Fig. 2A and Figs. S2A–C), which demonstrated a distinct separation between ARDS samples and non-ARDS control groups. A comprehensive differential expression analysis revealed that 1069, 319, and 511 proteins were uniquely altered in ARDS *vs.* HC, ARDS *vs.* DC, and DC *vs.* HC groups, respectively (Fig. 2B–C and Fig. S2D, as detailed in Additional file 2). These findings demonstrated the progressive features of serum protein alterations correlating with the severity of the disease. A critical subset of 214 differentially abundant proteins emerged as consistently regulated across ARDS, with 16 proteins showing persistent downregulation and 198 showing upregulation in comparisons of both ARDS *vs.* DC and ARDS *vs.* HC (Fig. 2F, Additional file 3). Among the downregulated proteins, Fetuin B (FETUB) can be considered a reliable biomarker for predicting mortality in *Staphylococcus aureus* bacteremia (SaB) patients because deceased FETUB levels correlate strongly with poor clinical outcomes in patients with SaB [10]. Paraoxonase 1 (PON1), negatively associated with higher mortality of sepsis [21], underlines significance in ARDS pathology compared to DC and HC groups (Fig. 2G). Conversely, certain proteins, including Surfactant Protein D (SFTPD) and Signal Transducers and Activators of Transcription 3 (STAT3) et al., were elevated (Additional file 3). Moreover, SFTPD, a circulating epithelial marker [22], and STAT3, an activator of macrophages and neutrophils [23], were implicated as potential key contributors to the pathogenesis of ARDS.

Pathway analysis identified nine distinct pathways that were specifically modulated in ARDS (Fig. 2D–E and Fig. S3). Notably, the oxidative phosphorylation pathway emerged as significantly upregulated in comparisons of ARDS with both DC (NES = 1.615, *P* = 0.001) and HC (NES = 1.582, *P* < 0.001), revealing its pivotal role in the energetic metabolism associated with ARDS (comprehensive pathway listings are available in Additional file 4 and Supporting Information). Fig. 2H illustrated the enriched proteins within these nine overlapping pathways, all exhibiting increased serum levels in ARDS cases. Among the highlighted proteins, Succinate Dehydrogenase Complex Iron-Sulfur Subunit B (SDHB), involved in mitochondrial function, and various components of the vacuolar ATPase family (ATP6V1D, ATP5MG, ATP6V1H) were implicated in oxidative phosphorylation. Moreover, proteins such as Mitogen-Activated Protein Kinase Kinase 1 (MAP2K1), Phosphoinositide-3-Kinase Regulatory Subunit 1 (PIK3R1), and Non-Receptor Tyrosine Kinase (SRC), associated with the VEGF signaling pathway, were significantly upregulated in ARDS, illustrating a comprehensive network of molecular interactions contributing to the pathogenesis and progression of ARDS.

### Cross‑talk between proteomics and metabolomics implicates the sphingolipid signaling pathway as a mediator in ARDS

**Fig. 3:**
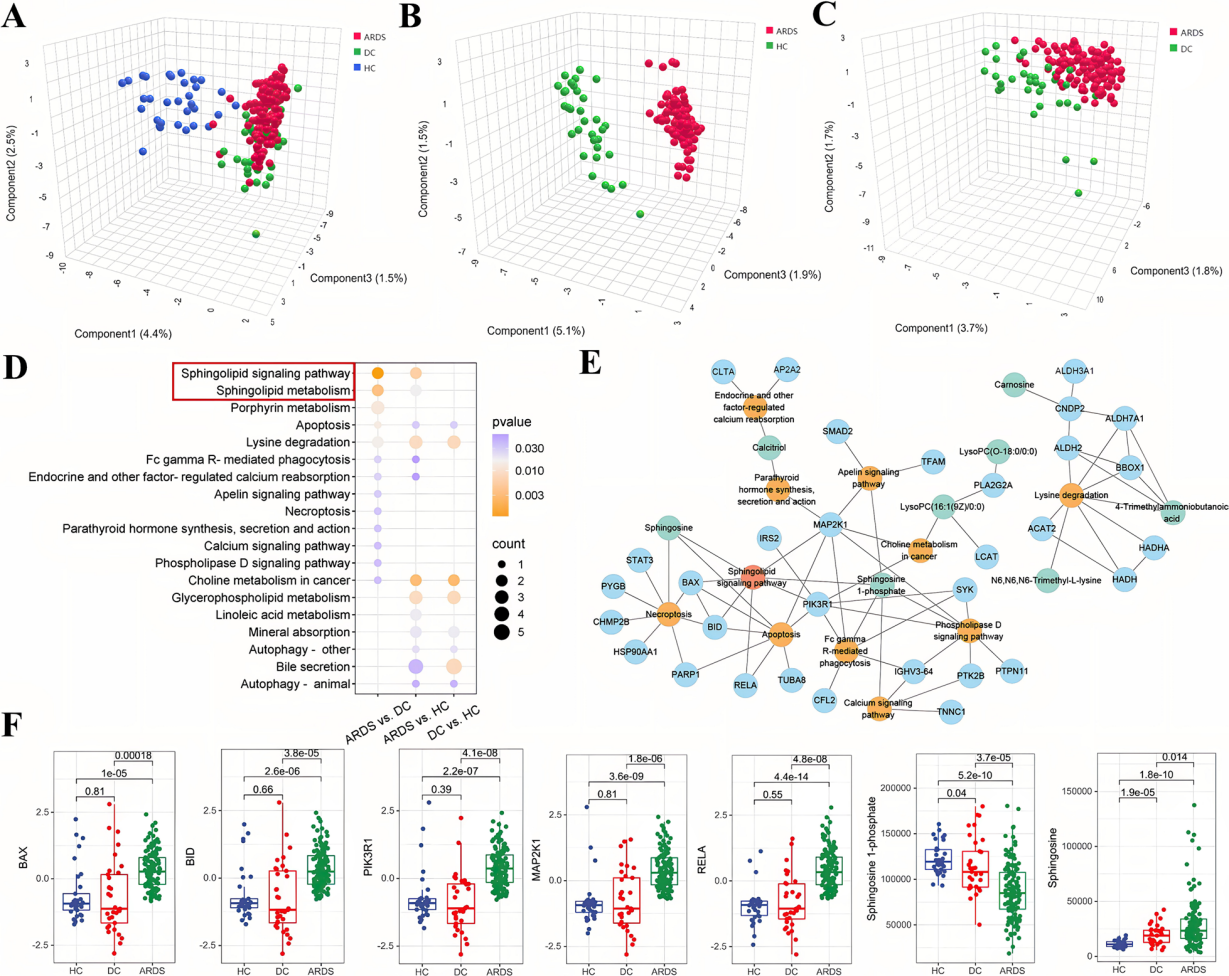
The metabolic analysis and its interaction with proteomics in ARDS in the discovery cohort. **A–C** Partial least squares discriminant analysis (PLS-DA) showed the separation of ARDS patients from HC and DC groups. **D** Dot plot of enriched KEGG pathways identified by differentially abundant metabolites. Dot size indicates the number of enriched proteins, colors represent the significance of enrichment (*P*-value). **E** Cross-talk network of the significantly enriched pathway by integrating both proteomics and metabolomics. Blue dots represent proteins, green dots are metabolites, and orange dots are enriched pathways. Sphingolipid signaling pathway was highlighted in red. **F** The relative abundance of proteins and metabolites in sphingolipid signaling pathway across ARDS, HC, and DC groups

In our metabolic investigation, we cataloged a comprehensive array of 3331 metabolites, encompassing amino acids, lipids, and other critical serum metabolites, as detailed in Additional file 5. The PCA plots revealed a pronounced metabolic differentiation of the ARDS in comparison to both DC and HC groups (Fig. 3A–C). We identified 214, 113, and 206 differentially abundant metabolites (DAMs) in the ARDS* vs.* HC, ARDS *vs.* DC, and DC *vs*. HC, respectively (Additional file 6). Among these, lysophosphatidylcholine (LysoPC) emerged as the most markedly altered metabolites in ARDS, exemplified by a significant decrease in LysoPC (0:0/18:2(9Z, 12Z)) and LysoPC (0:0/18:0). Further exploration through the KEGG pathway notably highlighted the modulation of the sphingolipid signaling pathway and sphingolipid metabolism, which were distinctive in ARDS as compared to the non-ARDS groups (Fig. 3D). Importantly, Fig. 3E revealed that metabolite S1P linked the sphingolipid signaling pathway with several other signaling pathways, including the Apelin, calcium, and phospholipase D signaling pathways. Additionally, S1P levels were reduced, whereas sphingosine exhibited elevated levels in ARDS compared to both DC and HC groups (Fig. 3F).

We further conducted an integrated analysis of proteomics and metabolomics to elucidate the pathogenetic mechanisms underlying ARDS. A significant finding from our study was the identification of the sphingolipid signaling pathway as a key regulatory axis, influenced at both the protein and metabolite levels, pinpointing it as a hub pathway in ARDS pathogenesis (Fig. 3D and Table S2). Further network analysis demonstrated the critical role of the sphingolipid signaling pathway, positioning it centrally within the ARDS-specific regulatory network (Fig. 3E and Figs. S4–S5). Specifically, critical regulatory proteins such as BCL2 Associated X (BAX), BH3 Interacting Domain Death Agonist (BID), PIK3R1, MAP2K1, and NF-κB Subunit (RELA) were upregulated in ARDS (Fig. 3F). Furthermore, MAP2K1, the hub protein in the network, linked the sphingolipid signaling pathway with several important pathways, including parathyroid hormone synthesis secretion and action, Apelin signaling pathway, choline metabolism, phospholipase D signaling pathway, Fc gamma R-mediated phagocytosis, and apoptosis. BAX and BID mediate interactions linking the sphingolipid signaling pathway with necroptosis and apoptosis, while RELA associates with the pathway in apoptosis. PIK3R1 connects the sphingolipid signaling pathway to Fc gamma R-mediated phagocytosis, apoptosis, phospholipase D signaling pathway, and choline metabolism (Fig. 3E).

### Dysregulation of biological functions and alterations in metabolites LysoPCs and S1P in deceased patients at ARDS onset

**Fig. 4:**
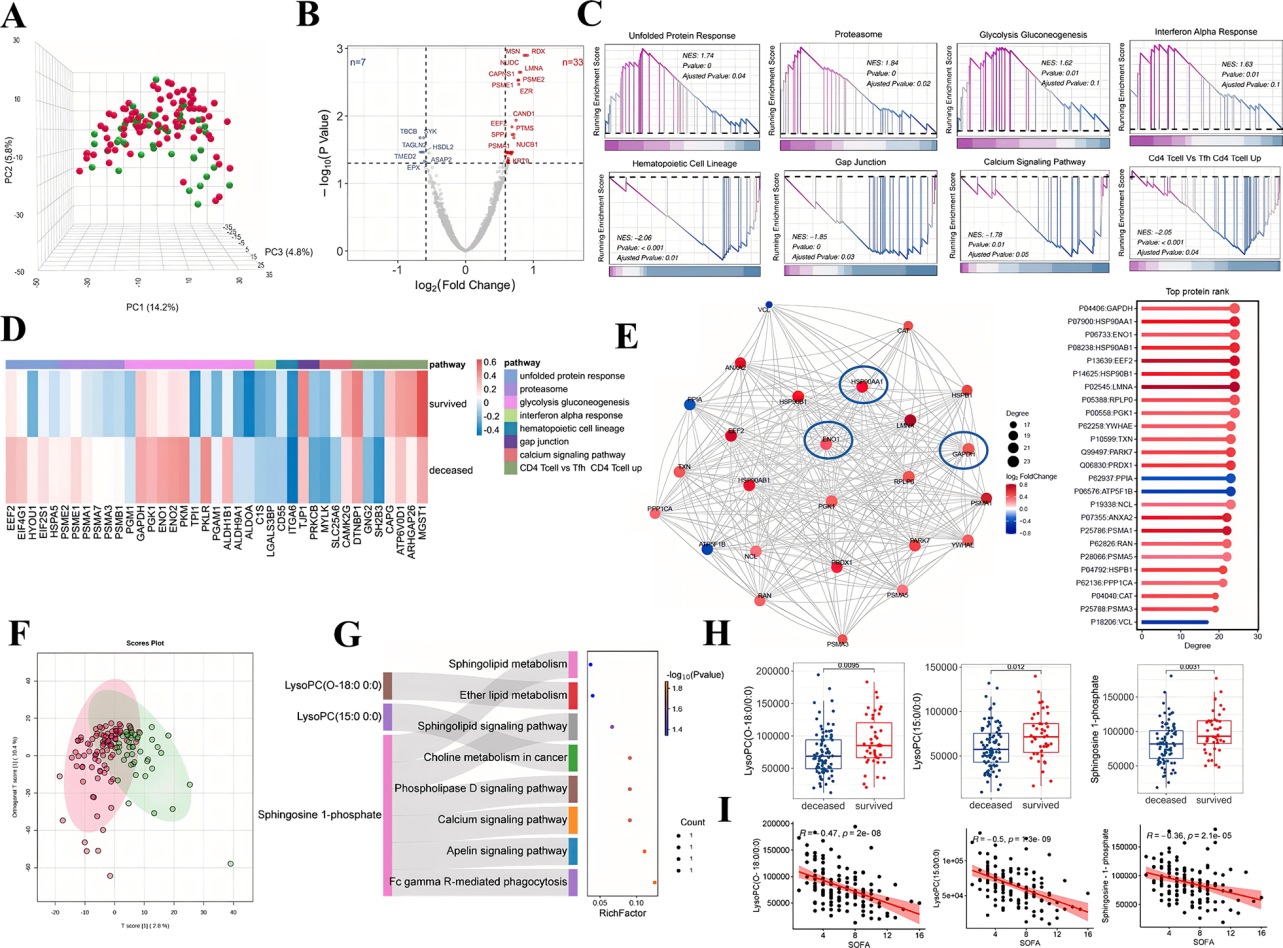
The multi-omics analysis compared deceased ARDS with survived ARDS.** A** PCA plot displays the proteome separation in deceased and survived ARDS. Each dot represents a sample, with red dots for deceased ARDS samples and green dots for survived ARDS samples. **B** Volcano plot displaying differentially abundant proteins between deceased and survived ARDS. *P* values were adjusted using the Benjamini–Hochberg method. **C** GSEA analysis showed up- and down-regulated pathways in deceased ARDS compared with survived ARDS. **D** A heatmap showed representative proteins linked to the pathways highlighted in **C**. The mean difference (deceased ARDS–survived ARDS) of proteins in pathways larger than 0.1 were kept. **E** Left: PPI network analysis of DAPs between deceased ARDS and survived ARDS. The significance threshold was defined as *P* < 0.05. The sliding ball chart on the right shows the network degree and log2FC of the corresponding proteins in the PPI network. (**F**) PLS-DA score plots between deceased and survived ARDS for metabolomics. **G** The Sankey diagram showed the enriched pathways and their associated DAMs. **H** The abundance of LysoPC (O-18:0/0:0), LysoPC (15:0/0:0), and S1P between deceased and survived ARDS groups. Statistical significance was determined using the FDR-adjusted *P*-value. *P* values were calculated by *Wilcoxon’s* rank sum test. **I** Correlations between metabolites mentioned in **H** and the SOFA scores. The correlation coefficients (simplified as *R*) and rho values of correlations are indicated

To further investigate the proteomic alterations associated with prognosis in ARDS, we conducted a comparative analysis between ARDS survivors and non-survivors (Fig. 4A). Differential expression analysis identified 40 proteins with significant variations (│fold change│≥ 1.5 and FDR < 0.05) (Table S3). The most significantly altered proteins between deceased and survived ARDS were Radixin (RDX) and Moesin (MSN), which play crucial roles in linking actin to the plasma membrane (Fig. 4B). Subsequent GSEA revealed several highly ranked molecular pathways markedly enriched across various biological functions such as energy metabolism, immune response, proteasome function, gap junction communication, calcium signaling, and hematopoietic cell lineage differentiation (Fig. 4C and Supporting Information). To refine our selection of key protein candidates, we spotlighted the top 25 proteins exhibiting the highest connectivity degrees in PPI network (Additional file 7). Among these, Glyceraldehyde-3-Phosphate Dehydrogenase (GAPDH), Heat Shock Protein 90 Alpha Family Class A Member 1 (HSP90AA1), and Enolase 1 (ENO1) emerged as central hub proteins within the network (Fig. 4D–E). This finding aligned with the GSEA results, as GAPDH and ENO1 were critical enzymes in glycolysis; moreover, HSP90AA1 was the housekeeping protein that aids protein folding and has intrinsic ATPase activity.

At the global metabolic level, the PCA plot demonstrated a slight separation between the ARDS-deceased and survived groups (Fig. 4F). The 45 DAMs were most enriched in signaling pathways, such as the sphingolipid signaling pathway, phospholipase D signaling pathway, calcium signaling pathway, and Apelin signaling pathway (Fig. 4G and Additional file 8). LysoPC (O-18:0/0:0), LysoPC (15:0/0:0), and S1P were prominently enriched in these pathways. Furthermore, the levels of LysoPC and S1P were significantly reduced in the ARDS-deceased group (Fig. 4H). More importantly, correlation analysis between these three metabolites and the severity of ARDS, as quantified by the SOFA score, revealed negative correlations (*R* = − 0.47 for LysoPC (O-18:0/0:0); *R* = − 0.50 for LysoPC (15:0/0:0); *R* = − 0.36 for S1P) (Fig. 4I), suggesting the potential metabolome mechanism underlying the heightened mortality risk in ARDS non-survivors.

### Biomarker panel for early prediction of ARDS prognosis

**Fig. 5:**
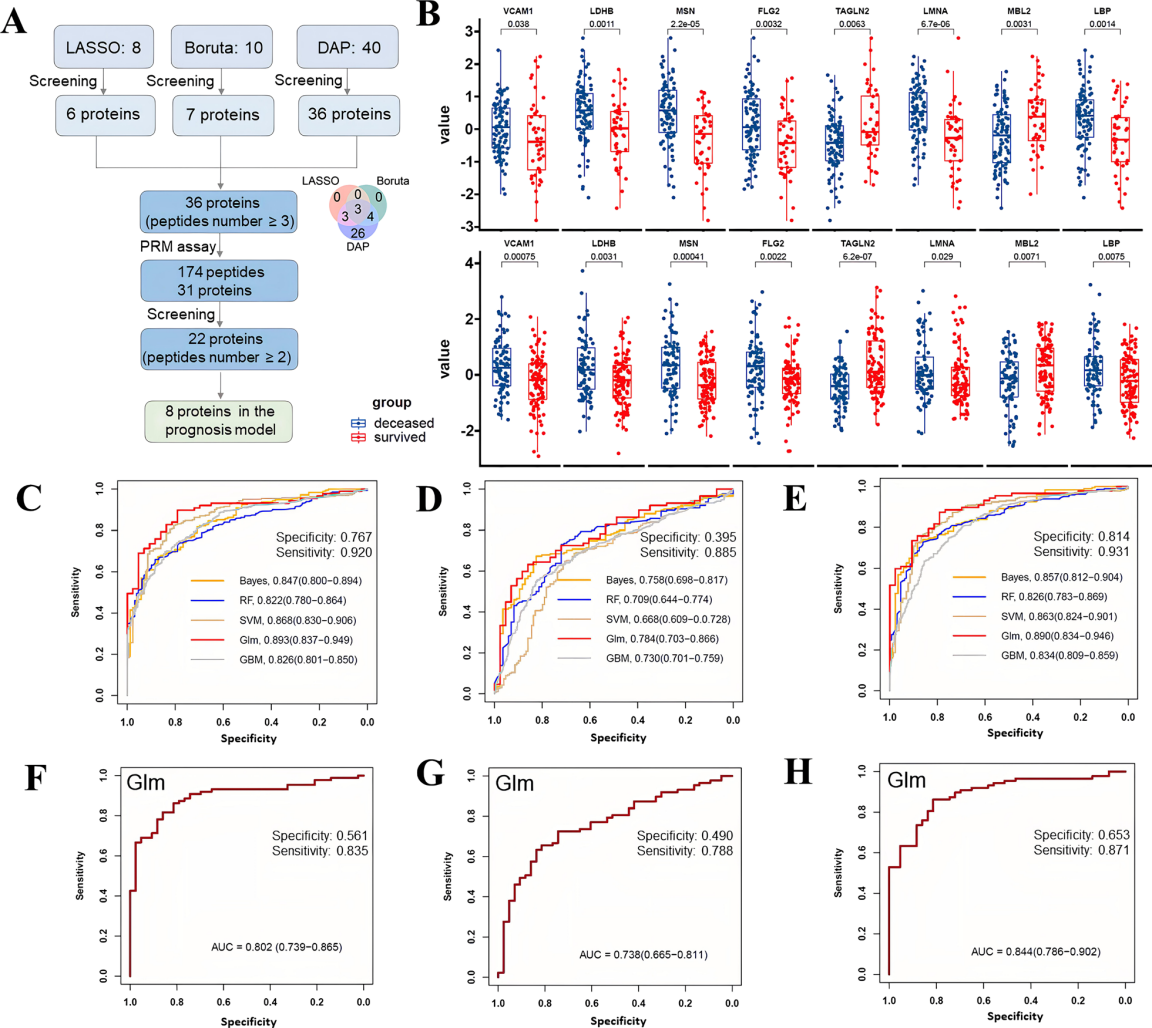
Prognostic model development and validation for ARDS. **A** The flowchart details the methodology for screening candidate biomarkers. **B** The expression levels of eight proteins between the deceased and survived ARDS in both discovery (the upper panel) and validation cohorts (the lower panel). Receiver operating characteristic curve of protein **C**, clinical **D**, and combined model **E** in the discovery cohort and the validation cohort **F–H**. The 95% confidence interval is shown between brackets. The sensitivity and specificity of the Glm model were also provided. AUC, area under the curve

Further, a prognosis model was constructed to predict the outcome of ARDS early. We obtained 36 candidate biomarkers using LASSO (Fig. S6A–B), Boruta, and DAPs. Finally, 31 proteins with 174 peptides were targeted by PRM assay in the external validation cohort (Additional file 9), and a total of 22 candidate proteins with peptides ≥ 2 were retained, in which 8 proteins maintained consistent significance in both discovery and validation cohorts (Fig. 5A–B). Among the eight proteins, six proteins were significantly upregulated in deceased patients in both discovery and validation cohorts, including Vascular Cell Adhesion Molecule 1 (VCAM1), Lactate Dehydrogenase B (LDHB), MSN, Filaggrin 2 (FLG2), Lamin A/C (LMNA), and Lipopolysaccharide Binding Protein (LBP), while two proteins, Transgelin 2 (TAGLN2) and Mannose Binding Lectin 2 (MBL2) were consistently downregulated in the deceased ARDS group (Fig. 5B). These markers were selected as an eight-protein panel for early identification of deceased patients of ARDS.

In the discovery cohort, we first compared five state-of-the-art machine learning classifiers using the protein model. The Glm model was the final classifier due to its overall superior performance, evidenced by a 10-fold cross-validated ROC–AUC of 0.893 (95% CI 0.837–0.949) and a sensitivity of 0.920 (Fig. 5C). In comparison, the clinical risk model, comprising the SOFA score, P/F ratio, and PLT from LASSO feature selection (Fig. S6C–D), yielded in a ROC-AUC of 0.784 (95% CI 0.703–0.866) and a sensitivity of 0.885 (Fig. 5D). Combination of parameters in both models resulted in a ROC-AUC of 0.890 (95% CI 0.834–0.946) and a sensitivity of 0.931 (Fig. 5E). The protein model performed significantly better than the clinical risk model (Delong test, *P* < 0.001), whereas the combination of both models did not exhibit statistical superiority over the protein model alone (Delong test, *P* = 0.970). Subsequent validation of the Glm classifier using the protein model resulted in a ROC-AUC of 0.802 (95% CI 0.739–0.865) and a sensitivity of 0.835 (Fig. 5F) in the external cohort, whereas the clinical risk model resulted in a ROC-AUC of 0.738 (95% CI 0.655–0.811) and a sensitivity of 0.788 (Fig. 5G). The combination of both models led to a ROC-AUC of 0.844 (95% CI 0.786–0.902) (Fig. 5H). In the validation cohort, the protein model also outperformed the clinical risk model (Delong test, *P* = 0.008), and a combination of both models was superior to the protein model alone (Delong test, *P* = 0.006).

## Discussion

This was the first study to integrate proteomics and metabolomics to investigate differences in the proteome, metabolome, and related biological pathways in adult ARDS patients. Our findings identified eight proteins as early candidates for distinguishing deceased ARDS cases from survivors. These candidate proteins, along with the constructed prognostic model, were validated in an independent external cohort, revealing their potential utility in clinical prognosis.

This integrative analysis offers a global perspective on the proteomic and metabolic landscapes of ARDS, highlighting the potential involvement of the sphingolipid signaling pathway, specifically the role of S1P, a molecule synthesized through the phosphorylation of sphingosine. Indeed, S1P was a sphingolipid that helped improve glycocalyx integrity by inhibiting syndecan-1 shedding [24]. S1P activated the S1P1 receptor, which attenuated the activity of metalloproteinases causing syndecan-1 ectodomain shedding [24, 25]. In addition, previous studies have suggested that S1P played a significant role in regulating vascular permeability, barrier integrity, and inflammation in ARDS [26–32]. S1P was protective against acute lung injury by regulating endothelial cell barrier integrity through binding to S1P receptors [30, 31, 33]. Murine and canine models revealed that S1P exerted potent beneficial systemic effects, significantly reducing vascular leak, LPS-induced lung edema formation, and inflammatory lung injury [28, 29]. Therefore, targeting the S1P or S1P1 receptor activation may be a potential therapeutic option for inhibiting glycocalyx degradation in ARDS. Similarly, decreased serum S1P levels have been associated with septic shock severity [34] and a significant association was identified between sphingolipid metabolism and outcomes in trauma patients [35]. These findings indicated that lower S1P levels correlated with increased disease severity in ARDS, although our study has not established mechanistic data.

In addition, in our study, ARDS patients exhibited higher levels of MAP2K1 (i.e., MEK1). MAP2K1, involved in the sphingolipid signaling pathway, was identified as the hub protein in the proteome-metabolome cross-talk network. Furthermore, Jonathan et al. found S1P signals regulated lymphatic endothelial cells permeability and junction molecule expression through MAPKs [36]. Meanwhile, MEK1 has been implicated in various signaling networks that affect tumor necrosis factor (TNF) mediated antiapoptotic signaling [37] and inflammatory profile [38]. Therefore, MEK inhibition has important therapeutic implications for ARDS by potentially disrupting the sphingolipid signaling pathway and its associated biological processes.

Notably, the concentrations of LysoPC species such as LysoPC (O-18:0/0:0) and LysoPC (15:0/0:0) in the deceased ARDS groups were decreased compared with the survived group. LysoPC is a lipid mediator derived from membrane phosphatidylcholine, and it is known to contribute to inflammation by increasing chemokine production and activating endothelium, neutrophils, monocytes, macrophages, and lymphocytes [39]. However, the role of LysoPCs in ARDS has not yet been clearly elucidated. In sepsis patients, serum LysoPC levels were lower than those in controls, and among them, LysoPC (16:0) and LysoPC (18:0) were decreased [40]. Lower concentrations of serum or plasma LysoPC predicted worse outcomes [41]. Our findings align with these observations, revealing a comparable reduction in serum LysoPC levels in ARDS, akin to that observed in sepsis. Therefore, LysoPCs could be important metabolite markers for differentiating the diagnosis and prognosis of ARDS, which needs further validation and mechanism exploration.

Moreover, our analysis presented a comprehensive view of proteomic alterations distinguishing deceased ARDS from survived ARDS, the elucidation of the functional roles and molecular mechanisms of these proteins in ARDS lays the groundwork for potential therapeutic innovations targeting energy metabolism, such as glycolysis. One previous study showed that the increase of proinflammatory cytokines including pro-IL-1β and TNF-α preceded the activation of glycolysis in macrophages during LPS-induced ALI, suggesting that inflammation may induce glycolysis [42]. In COVID-19 ARDS, T cells are exhausted and skewed towards glycolysis, with a concomitant reduction in mitochondrial dependence, probably as a result of reduced oxygenation of the pulmonary tissue [43, 44]. Consistently, we identified upregulated glycolysis, interferon-α response, and activated CD4^+^ T cells in ARDS, indicating that glycolysis is coordinated with inflammation, beyond just generating energy and producing building blocks for cellular survival and signal transduction [45].

In this study, our serum biomarkers and prognosis model could indeed be a promising prognosis predictor of ARDS patients compared to traditional clinical models. Therefore, we proposed that the protein model based on serum proteomics has shown promise in identifying high-risk populations for deceased ARDS. Interestingly, a previous study revealed VCAM1 overexpressed on endothelial cell-derived extracellular vesicles during sepsis, facilitating the activation of the NF-*κ*B pathway by interacting with integrin subunit alpha 4 (ITGA4) on the monocyte surface thereby regulating monocyte differentiation [46]. Furthermore, the biomarker MBL2 has been identified as a critical determinant of mortality risk among patients with severe pneumococcal infections who exhibit MBL deficiency [47]. ‘MBL deficiency’ appears to play a role in increasing the susceptibility to severe infections in patients receiving stem cell transplantation [48, 49], highlighting the pathogenic significance of this innate immune defense protein. Other candidate biomarkers, for example, LDHB was engaged in glycolysis [50], MSN was expressed in the endothelial cell and was important for cell-cell recognition and signaling and for cell movement [51]. LBP is the LPS binding protein, the LBP-LPS complex initiates a signal cascade that triggers the secretion of pro-inflammatory cytokines [52]. Abnormal LMNA results in nuclear structural abnormalities and mesenchymal tissue damage [53], which are critical in ARDS pathogenesis due to their roles in cellular integrity and repair mechanisms. FLG2 is involved in epidermal maturation [54], a process that may impact the barrier function of the alveolar epithelium in ARDS. TAGLN2 facilitating the formation of intracellular cytoskeleton structures [55], which are essential for maintaining cell shape and motility, and crucial in the inflammatory response seen in ARDS. These biomarkers can be categorized into several groups: epithelial injury, endothelial damage, imbalanced immune system, mitochondrial dysfunction, and disrupted cytoskeleton, which engaged in the overall ARDS progress. However, the specificity of these eight proteins for predicting ARDS prognosis should be confirmed through either singular or multiple assessments, including enzyme-linked immunosorbent assay (ELISA), proximity extension assay (PEA), or bead-based immunoassays, which would benefit its application in future clinical practice.

We acknowledge several limitations of the present study. First, while we identified eight proteins associated with adverse prognosis in ARDS, further studies are required to explore the underlying mechanisms by which these proteins influence disease outcomes, particularly focusing on their specific roles within the immune and pathology systems. Second, longitudinal multi-omics studies are needed to explore the dynamic features of ARDS and the temporal changes in prognostic biomarkers. Such studies will provide a more comprehensive understanding of the disease progression and the potential for early intervention. Third, integrating metabolomics and proteomics presents significant methodological challenges, such as data harmonization and handling missing values, which may introduce biases and affect the robustness of our findings. Lastly, the identification of intracellular metabolites or proteins in the circulation does not necessarily indicate changes in cellular or tissue processes. These findings may also reflect cell death and the release of intracellular contents, which should be taken into account when interpreting the results.

In conclusion, this comprehensive study highlights the importance of the sphingolipid signaling pathway in unraveling pathogenesis of ARDS. Protein MAP2K1 and metabolite S1P play critical roles in this pathway. We show that a panel of eight proteins is superior to a clinical prognostic model in predicting ARDS-deceased events. These findings have significant implications for risk assessment and potential guidance of therapeutic strategies in the management of ARDS.

### Supplementary Information

Additional file 1.

Additional file 2.

Additional file 3.

Additional file 4.

Additional file 5.

Additional file 6.

Additional file 7.

Additional file 8.

Additional file 9.

Additional file 10.

## Data Availability

Data and research materials used in this study are available upon request to qualified researchers for purposes of replication, further analysis, and academic collaboration.

## Abbreviations

ICU: Intensive care unit
S1P: Sphingosine 1-phosphate
PRM: Parallel reaction monitoring
DIA: Data-independent acquisition
PCA: Principal component analysis
DAP: Differentially abundant proteins
DAM: Differentially abundant metabolites
GSEA: Gene set enrichment analysis
LASSO: Least absolute shrinkage and selection operator
Bayes: Naïve Bayes
RF: Random forest
Glm: Generalized linear model
SVM: Supporting vector machine
GBM: Gradient boosting machine
PPI: Protein–protein interaction
FDR: False discovery rate
FC: Fold change
NES: Normalized enrichment score
ROC: Receiver operating characteristic
AUC: Area under the curve
