# Supplementary material for: Integrative multi-omics analysis unravels the host response landscape and reveals a serum protein panel for early prognosis prediction for ARDS
Source: Crit Care. 2024 Jul 2;28:213. doi: 10.1186/s13054-024-05000-3 (PMC11218270; doi:10.1186/s13054-024-05000-3)
Supplement: Supplementary file 10 — Additional file 10. [file 13054_2024_5000_MOESM10_ESM.docx]

**SUPPORTING INFORMATION**

**Integrative Multi-Omics Analysis Unravels the Host Response Landscape and Reveals a Serum Protein Panel for Early Prognosis Prediction for ARDS**

Mengna Lin^1,2†^, Feixiang Xu^2†^, Jian Sun^2^, Jianfeng Song^3^, Yao Shen^4^, Su Lu^2^, Hailin Ding^2^, Lulu Lan^2^, Chen Chen^2^, Wen Ma^5^, Xueling Wu^6,*^, Zhenju Song^2,1,7*^, Weibing Wang^1^^,5*^

^1^Shanghai Institute of Infectious Disease and Biosecurity，School of Public Health, Fudan University, Shanghai, China;

^2^Department of Emergency Medicine, Zhongshan Hospital, Fudan University, Shanghai, China;

^3^Department of Emergency Medicine, Minhang Hospital, Fudan University, Shanghai, China;

^4^Department of Respiratory Medicine, Pudong Hospital, Fudan University, Shanghai, China;

^5^School of Public Health, Fudan University, Shanghai, China;

^6^Department of Respiratory Medicine, Renji Hospital, Shanghai Jiaotong University School of Medicine, Shanghai, China;

^7^Institute of Emergency Rescue and Critical Care, Fudan University, Shanghai, China

^†^Mengna Lin and Feixiang Xu contributed equally.

^*^Correspondence: [wwb@fudan.edu.cn](mailto:wwb@fudan.edu.cn); [song.zhenju@zs-hospital.sh.cn](mailto:song.zhenju@zs-hospital.sh.cn); [wuxueling76@126.com](mailto:wuxueling76@126.com)

**Methods**

**Proteomics sequencing and data preprocessing**

After magnetic separation, beads were washed with Reagent G and incubated with serum at 37°C for 1 hour. Post-incubation, beads were thrice washed with Reagent G to retain enriched proteins. For digestion, beads were treated with Reagent A, followed by heating with Reagent B at 95°C for 5 minutes. Upon returning to room temperature, the mixture was digested enzymatically with Reagents C and D at 37°C for 2 hours. Digestion was terminated using Reagent E, and the supernatant was collected post-centrifugation for desalting. Peptide desalting involved activating a desalting column with methanol, conditioning with Condition Buffer, and equilibrating with Wash Buffer, each step followed by centrifugation. The peptides were loaded onto the column, washed, and eluted with Elution Buffer, resulting in a 60 µL concentrated sample. Before mass spectrometry, each sample was mixed with iRT standard (Biognosys, ThermoFisher) in a volume ratio of 1:20 (iRT: sample). The iRT standard kit, comprising 11 synthetic peptides not found in nature, is designed for optimized stability, sensitivity, and retention time, making it an ideal internal standard for chromatographic calibration and quantitative quality control without influencing the test samples.

**Metabolomics sequencing and data preprocessing**

For the obtained metabolomic data, we applied a series of quality control and pre-processing steps to ensure data reliability and accuracy. Ion peaks with missing values (0 value) greater than 50% within any group were excluded from further analysis. The remaining 0 values were replaced with half of the minimum value detected across all samples. Compounds were screened based on their qualitative result scores. Compounds scoring less than 36 points (out of a maximum of 80 points) were considered to have inaccurate qualitative results and were removed from the dataset. A QC sample was prepared by mixing aliquots of all samples. This QC sample was used to assess the reproducibility and stability of the analytical process. Principal Component Analysis (PCA) was used to visualize metabolic alterations among different samples[[1](#_ENREF_1)]. A data matrix was combined from the positive and negative ion data. Partial Least-Squares-Discriminant Analysis (PLS-DA) was utilized to distinguish the metabolites that differ between groups. To prevent overfitting, 7-fold cross-validation and 200 Response Permutation Testing (RPT) were used to evaluate the quality of the model.

Variable Importance of Projection (VIP) values obtained from the OPLS-DA model were used to rank the overall contribution of each variable to group discrimination. A two-tailed Student’s T-test was further used to verify whether the metabolites of difference between groups were significant. Differential metabolites were selected with VIP values greater than 1.0 and p-values less than 0.05.

**PRM assay**

To evaluate the candidate biomarkers of prognosis, a unique PRM assay was generated, incorporating as many candidate proteins as possible. For the proteome profiling samples, the peptide was examined utilizing a Q Exactive HF-X Hybrid Quadrupole-Orbitrap Mass Spectrometer (Thermo Fisher Scientific), integrated with a state-of-the-art high-performance liquid chromatography system (EASY nLC 1200, Thermo Fisher Scientific). The peptide samples, once dried, were reconstituted in Solvent A (0.1% formic acid in water). These reconstituted samples were then loaded onto a 2-cm, self-packed trapping column (with an inner diameter of 100 μm and packed with 3 μm ReproSil-Pur C18-AQ beads from Dr. Maisch GmbH) using Solvent A. Subsequently, separation was achieved on a column of 150 μm in inner diameter and 15 cm in length (1.9 μm ReproSil-Pur C18-AQ beads from Dr. Maisch GmbH). This separation process spanned 150 minutes, utilizing a gradient composed of Solvent A and Solvent B (0.1% formic acid in 80% ACN) at a constant flow rate of 600 nL/min (0–30 min, 0 min, 8% B; 0–4 min, 8–15% B; 4–19 min, 15–30% B; 19–22 min, 30–50% B; 23–24 min, 50–100% B; 24–30 min, 100% B). Ionization of the eluted peptides was achieved at 2 kV, after which they were introduced into the mass spectrometer. The instrument operated in a Parallel Reaction Monitoring acquisition mode, capturing full scan MS1 spectra of ions with a mass-to-charge ratio (m/z) ranging from 300 to 1400, utilizing the Orbitrap mass analyzer set to a high resolution of 60,000. The automatic gain control (AGC) target was established at 3E+06, with a maximum ion injection time of 80 ms. A selection of 20 precursor ions was made for fragmentation in the HCD cell, employing a normalized collision energy of 27%. The resultant fragment ions were analyzed by the Orbitrap at a resolution of 15,000, with the AGC for MS/MS set to 1e6 and the maximum ion injection time maintained at 80 ms.

Data processing of the PRM results was conducted using Skyline software. This involved sequential importation of the spectral library and PRM detection data. Parameters set during the import of spectral library data included a false discovery rate (FDR) of 1%, a precursor ion length range of 6-25 amino acids, charge states of 2 or 3 for precursor ions, and 1 or 2 for fragment ions, with a selection of y, p, and b ion types for fragment ions. The ion mass tolerance was set to 0.05 Da, and a maximum of 7 fragment ions were analyzed. The quantification results for targeted ions were exported for further analysis.

**Results**

**Altered serum proteomic profiling in the ARDS group and the biological pathways**

In addition to oxidative phosphorylation, key biological processes such as the spliceosome and proteasome pathways were upregulated in ARDS (Spliceosome: ARDS *vs.* DC, NES = 1.474, *P* = 0.007; ARDS *vs.* HC, NES = 1.500, *P* < 0.001. Proteasome: ARDS *vs.* DC, NES = 1.471, *P* = 0.011; ARDS *vs.* HC, NES = 1.534, *P* = 0.003). Signaling pathways, including mTOR, FoxO, VEGF, and sphingolipid signaling, also showed significant upregulation in ARDS relative to both DC and HC, indicating the complexity of molecular alterations in ARDS, including immune system, cell signaling and lipid mechanism. An exploration of unique pathways between ARDS and HC revealed an upregulation of processes related to amino acid degradation, autophagy, endocytosis, and the TNF signaling pathway, alongside restricted focal adhesions in ARDS (**Fig. 2D** and **Fig. S2**).

**Dysregulation of biological functions and alterations in metabolites LysoPCs and S1P in deceased patients at ARDS onset**

Remarkably, pathways like the unfolded protein response (NES = 1.74, *P* = 0.04), proteasome (NES = 1.84, *P* = 0.02), glycolysis/gluconeogenesis (NES = 1.62, *P* = 0.1), and interferon-α response (NES = 1.63, *P* = 0.1) showed positive associations with the deceased ARDS group. Conversely, pathways associated with hematopoietic cell lineage (NES = -2.06, *P* = 0.01), gap junctions (NES = -1.85, *P* = 0.03), calcium signaling (NES = -1.78, *P* = 0.05), and CD4^+^ T cell activity (NES = -2.05, *P* = 0.04) were more prominent in patients who survived ARDS in the early phase.


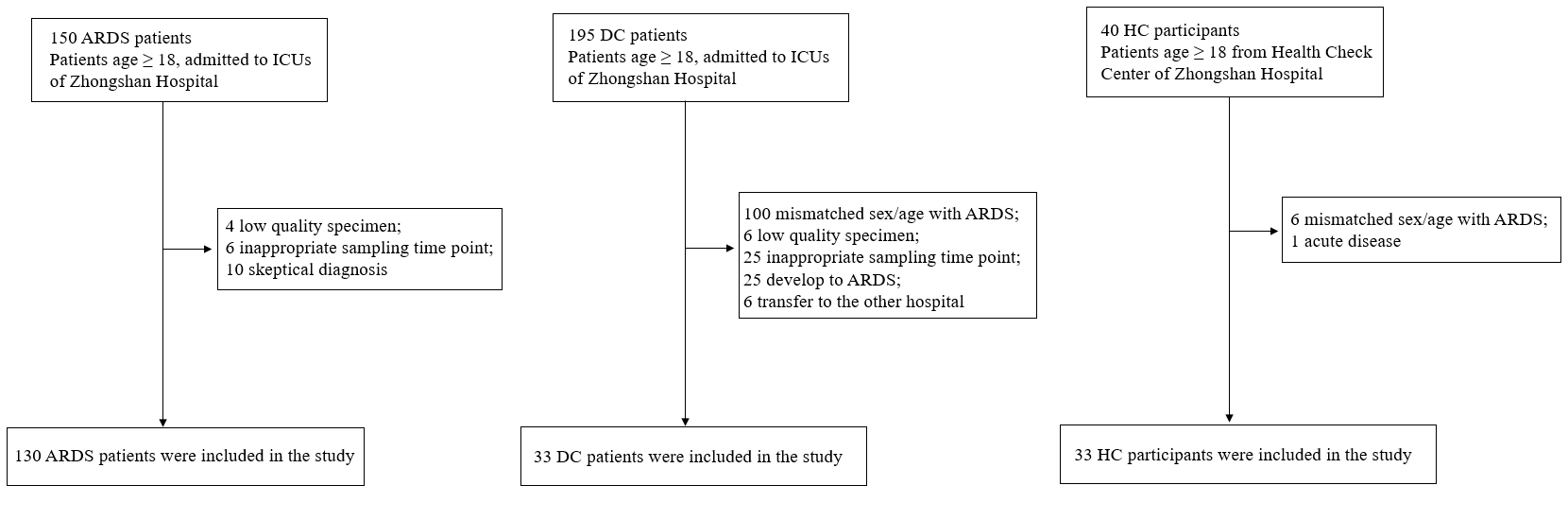


**Figure S1.** The overall flowchart of the discovery cohort.


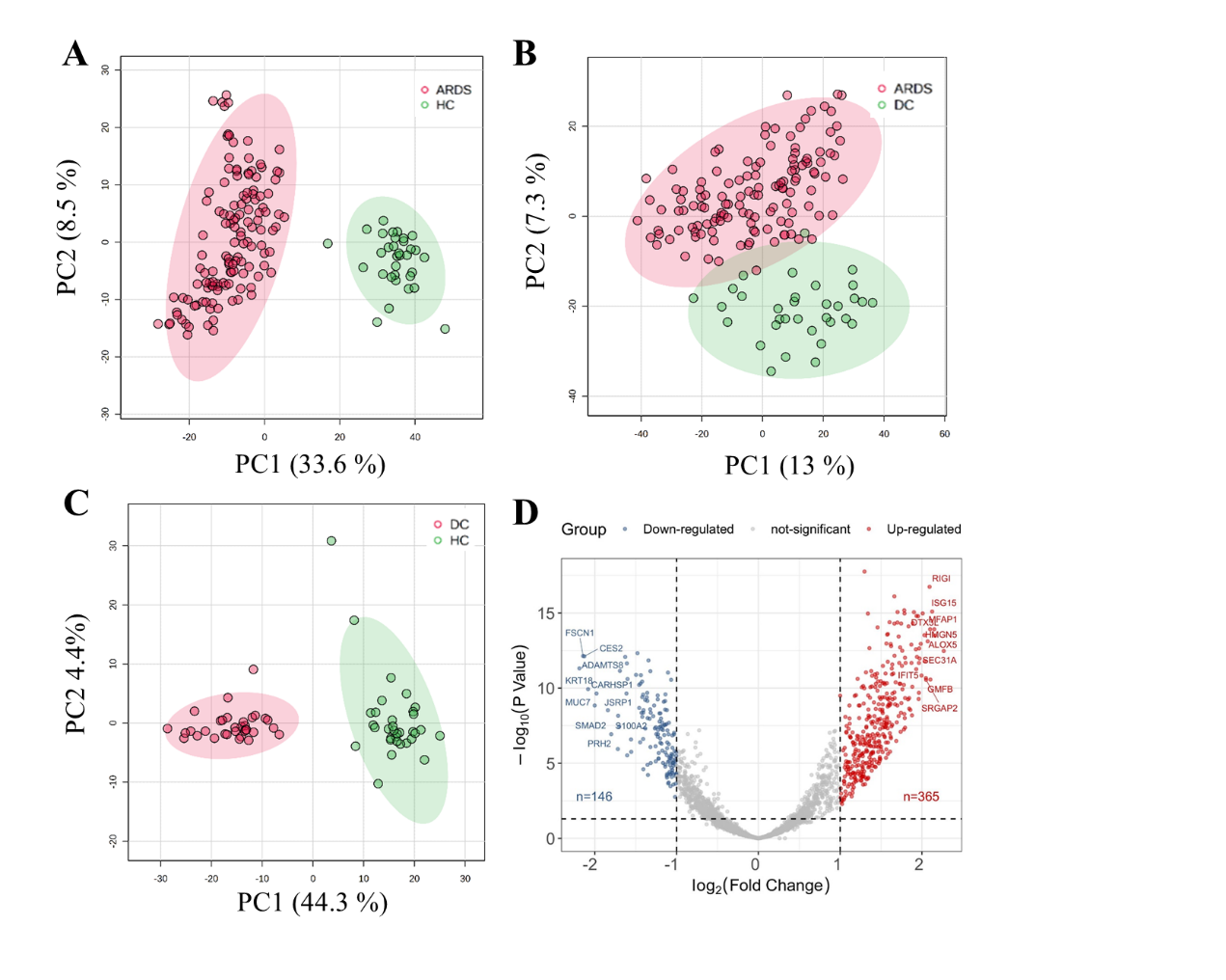


**
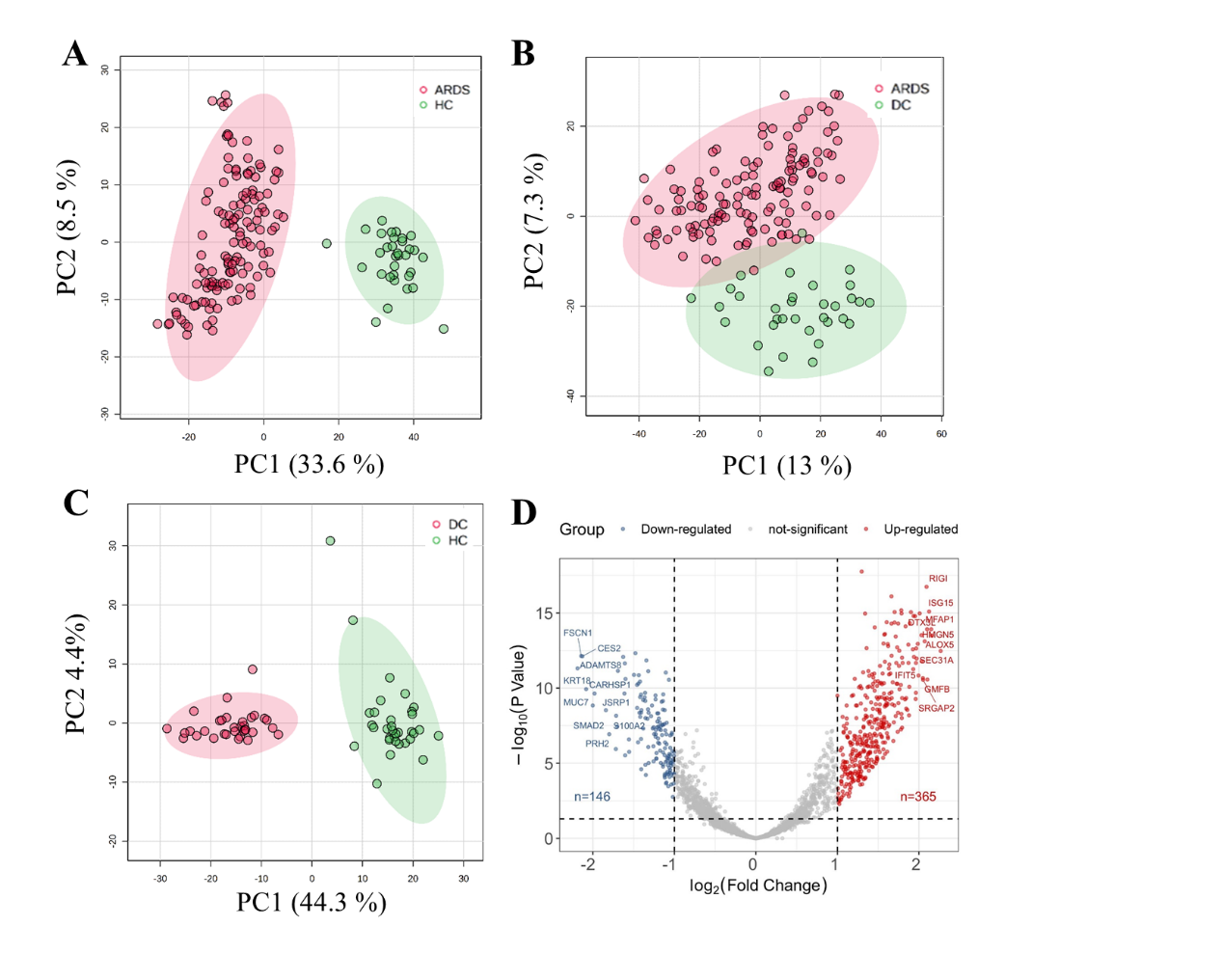
**

**Fig. S2** (**A-C**) The PCA plots across ARDS, DC and HC groups. (**D**) The volcano plot of differentially abundant proteins when DC group compared with HC group.

**
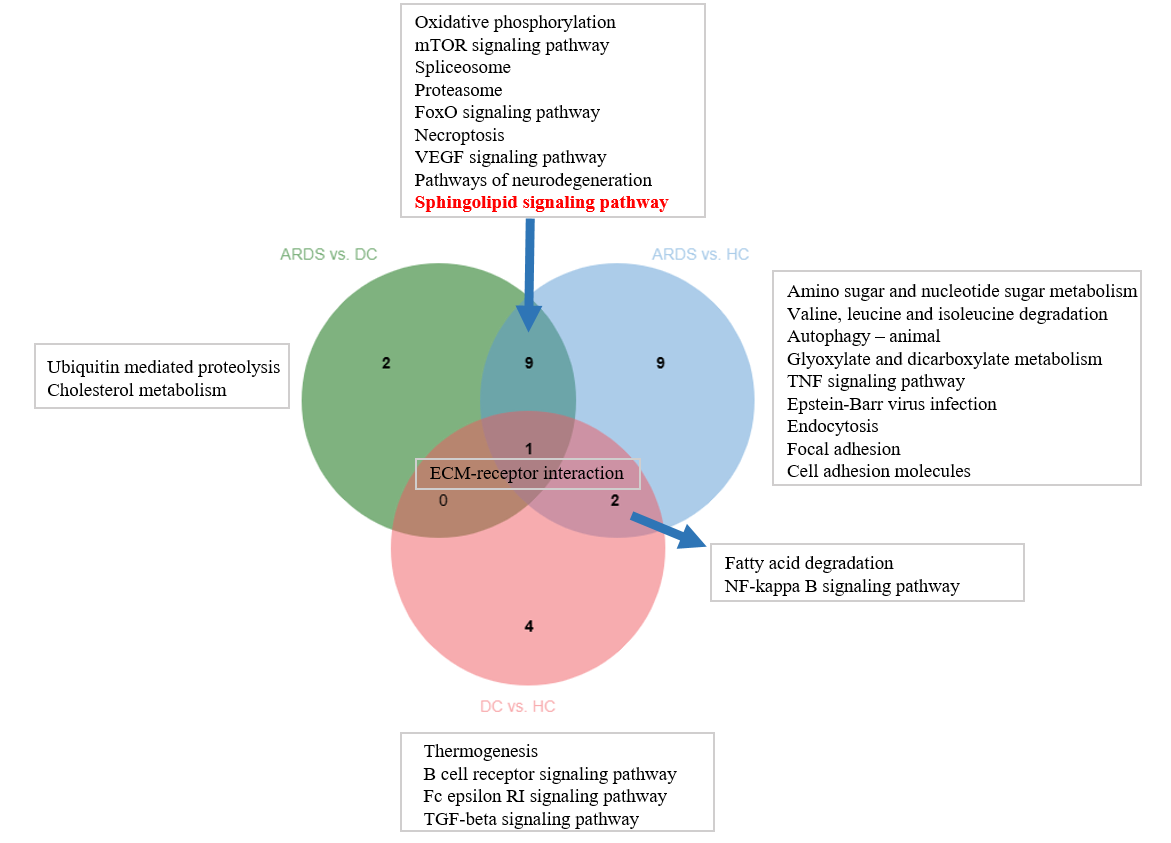
**

**Fig. S3** The Venn plot for pathways.

**
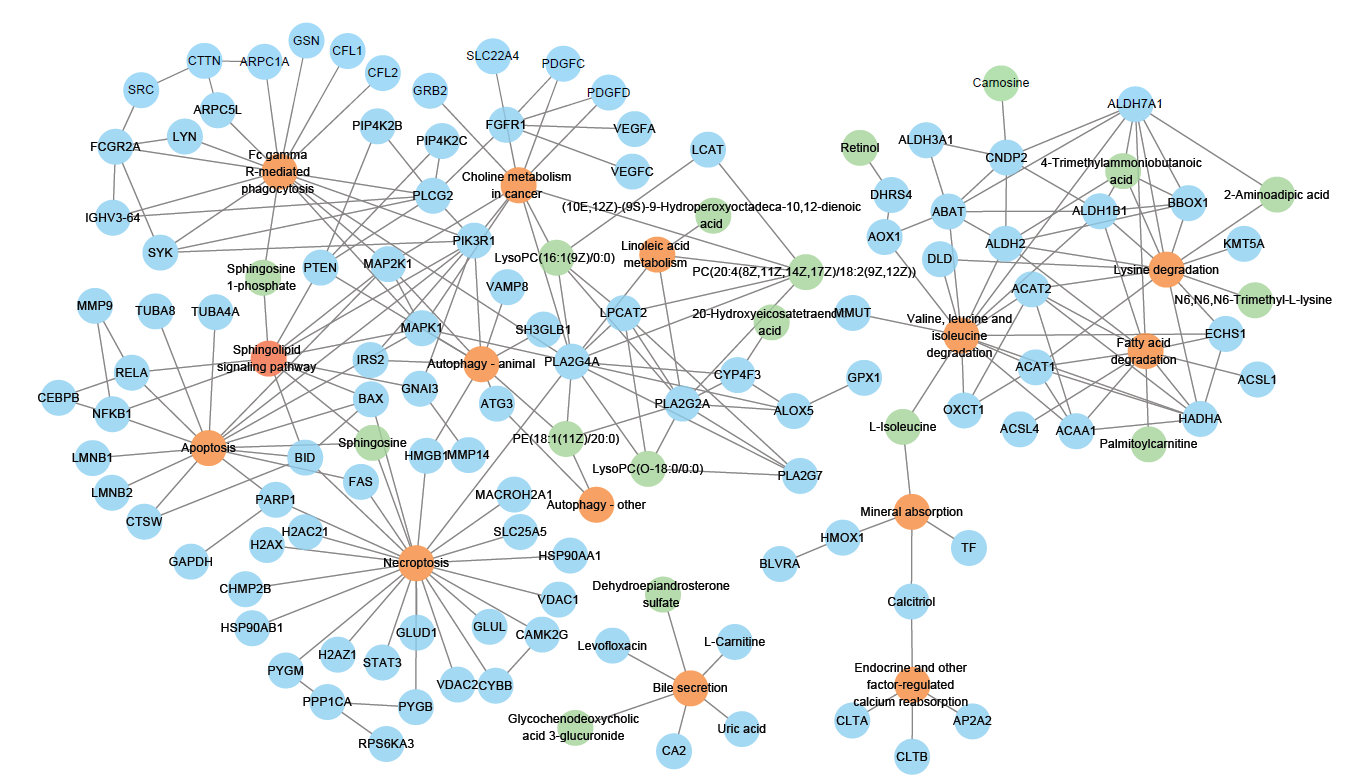
**

**Fig. S4** The cross-talk network in ARDS compared HC group.

**
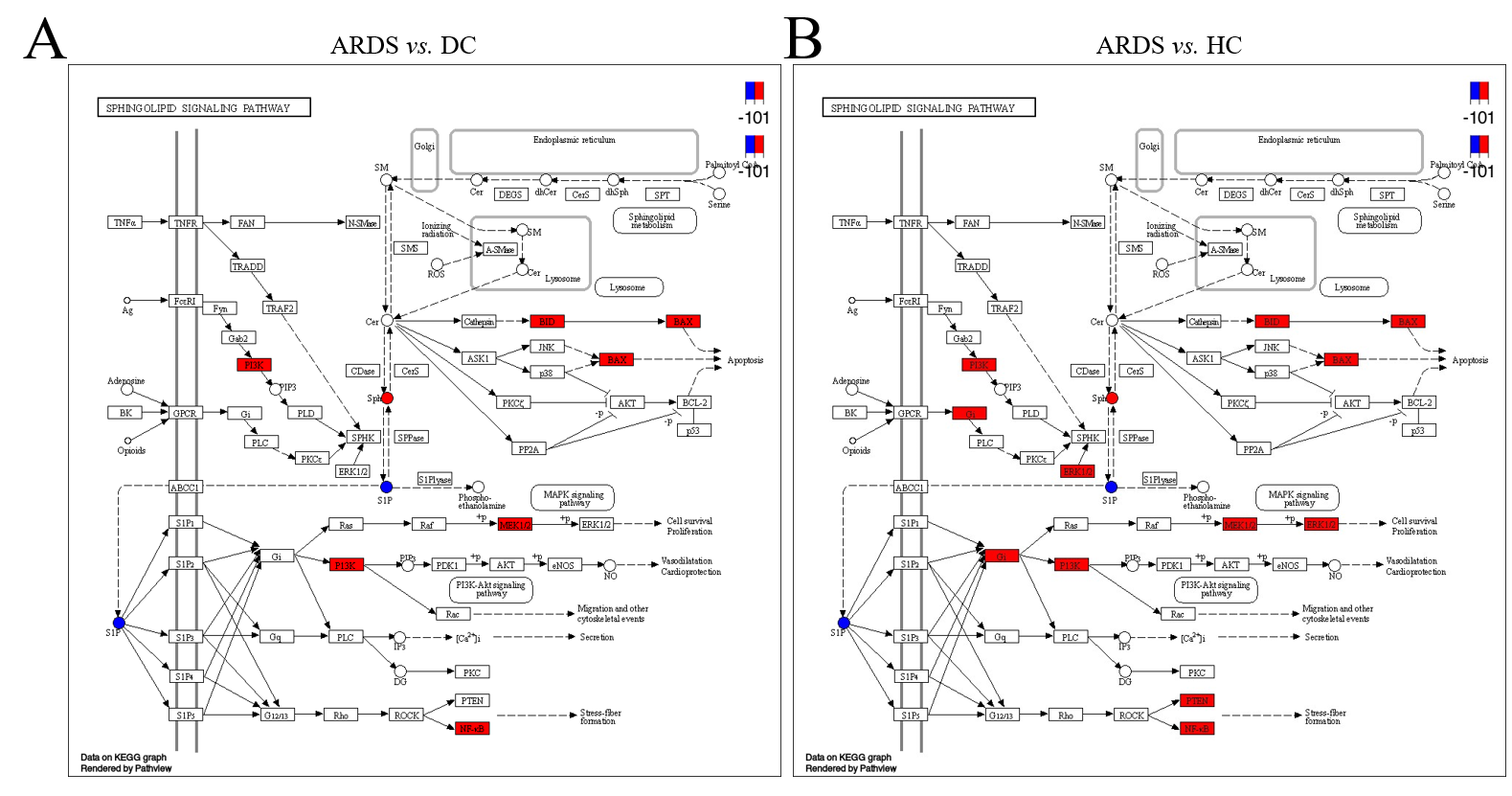
**

**Fig. S5** The protein-metabolite network in ARDS *vs.* DC (**A**) and ARDS *vs.* HC (**B**).

**
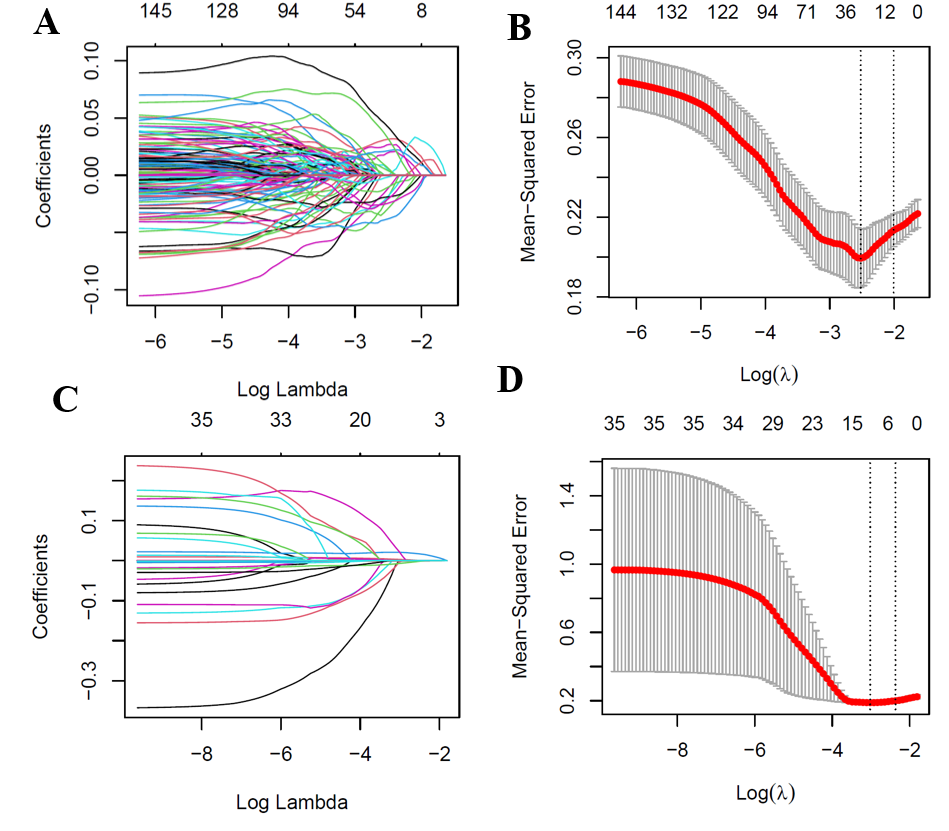
**

**Fig. S6** (**A-B**) The feature selection to screen the candidate protein biomarkers based on the proteomics. (**C-D**) The feature selection to screen the candidate parameters in the clinical prognosis model. (**A, C**) LASSO regression coefficient selection and variable screening. The lower horizontal axis represents lambda value, and the upper horizontal axis scale represents the number of variables in the LASSO regression model. **(B, D)** Cross-validation in the LASSO regression model to select the tuning parameter. The horizontal axis represents the log (lambda) value, and the vertical axis represents partial likelihood deviance. Error bars represent the means ± SD (by 10-fold cross-validation). The minimum mean 10-fold cross-validated error of λ is selected.

**Table S1. The baseline characteristics of the validation cohort.**

|  | Survived ARDS (n=98) | Deceased ARDS (n=85) | | *P* value |
| --- | --- | --- | --- | --- |
| Age (mean, SD) | 71.9 (14.6) | 73.9 (12.7) | | 0.233 |
| Male (n, %) | 76 (77.6) | 58 (68.2) | | 0.157 |
| SOFA | 3 [2-6] | 5 [5-8] | | <0.001*** |
| P/F ratio | 186.5 [117.9-265.5] | 100.0 [68.5-210.0] | | <0.001*** |
| RBC (*10^12^/L) | 3.92 [3.27-4.47] | 3.91 [3.36-4.39] | | 0.338 |
| Hb (g/L) | 119.0 [97.0-135.8] | 111.0 [95.0-130.0] | | 0.143 |
| WBC (*10^9^/L) | 10.62 [7.23-14.03] | 10.34 [7.41-15.22] | | 0.911 |
| NEU (*10^9^/L) | 8.8 [6.0-11.8] | 9.9 [6.5-15.8] | | 0.076 |
| LYM (*10^9^/L) | 0.7 [0.5-1.1] | 0.6 [0.4-0.9] | | 0.06 |
| PLT (*10^9^/L) | 178.5 [134.0-232.3] | 135.0 [79.0-192.0] | | <0.001*** |
| ALT (U/L) | 30.5 [18.0-58.5] | 29.0 [18.0-41.0] | | 0.654 |
| AST (U/L) | 31.0 [20.0-51.5] | 37.0 [25.0-53.0] | | 0.152 |
| STB (µmol/L) | 10.95 [7.80-17.90] | 11.30 [9.15-22.80] | | 0.069 |
| CB (µmol/L) | 4.05 [2.58-5.98] | 4.20 [2.89-8.40] | | 0.231 |
| BUN (mmol/L) | 8.45 [5.86-12.25] | 10.40 [6.80-19.40] | | 0.003** |
| Cr (µmol/L) | 74.5 [62.3-98.3] | 83.0 [67.0-134.5] | | 0.029* |
| Alb (g/L) | 33.0 [29.0-35.3] | 31.0 [28.5-34.0] | | 0.057 |
| CRP (mg/L) | 52.6 [18.05-132.3] | 79.9 [29.0-148.1] | | 0.148 |
| PCT (ng/mL) | 0.21 [0.07-0.88] | 0.27 [0.14-1.27] | | 0.514 |
| Fg (mg/dL) | 448.0 [281.5-539.3] | 451.0 [207.0-516.0] | | 0.102 |
| PT (second) | 13.1 [12.3-14.2] | 14.0 [12.9-15.6] | | 0.153 |
| APTT (second) | 28.75 [25.6-32.7] | 29.8 [26.3-33.9] | | 0.798 |
| D-dimer (mg/L) | 3.00 [1.19-8.52] | 2.50 [1.31-6.92] | | 0.753 |
| Hypertension (n, %) | 49 (50.0) | 41 (48.2) | | 0.812 |
| Coronary heart disease (n, %) | 14 (14.3) | 22 (25.9) | | 0.05 |
| Diabetes (n, %) | 32 (32.7) | 25 (29.4) | | 0.638 |
| Cerebrovascular disease (n, %) | 13 (13.3) | 17 (20.0) | | 0.221 |
| Immunosuppression (n, %) | 12 (12.2) | 8 (9.4) | | 0.541 |
| Vasopressor use at enrolment (n, %) | 12 (12.2) | 22 (25.9) | | 0.030* |
| Neuromuscular blockade use at enrolment (n, %) | 28 (28.6) | 22 (25.9) | | 0.810 |
| Mechanical ventilation (n, %) | 85 (86.7) | 80 (94.1) | | 0.155 |
| Prone positioning at enrolment (n, %) | 40 (40.8) | 56 (65.9) | | 0.001** |
| Risk factors (n, %) |  |  | | 0.043* |
| Pneumonia | 72 (73.5) | 65 (76.5) | |  |
| Sepsis | 17 (17.3) | 19 (22.4) | |  |
| Other | 9 (9.2) | 1 (1.2) | |  |
| Source of infection (n, %) |  |  | | 0.086 |
| Thorax | 72 (73.5) | 65 (76.5) | |  |
| Abdomen | 16 (16.3) | 18 (21.2) | |  |
| Other | 10 (10.2) | 2 (2.4) |  | |

*P*: Comparison between survived ARDS and deceased ARDS. **P* < 0.05, ***P* < 0.01, ****P* < 0.001.

**Table S2. The pathway enrichment in metabolomics.**

| **group** | **Annotation** | **count** | **FDR** | **RichFactor** |
| --- | --- | --- | --- | --- |
| ARDS vs. DC | Sphingolipid signaling pathway | 2 | 0.001076 | 0.133333333 |
| ARDS vs. DC | Sphingolipid metabolism | 2 | 0.003516 | 0.074074074 |
| ARDS vs. DC | Porphyrin metabolism | 3 | 0.011874 | 0.02027027 |
| ARDS vs. DC | Apoptosis | 1 | 0.013473 | 0.25 |
| ARDS vs. DC | Lysine degradation | 2 | 0.014609 | 0.035714286 |
| ARDS vs. DC | Fc gamma R-mediated phagocytosis | 1 | 0.02678 | 0.125 |
| ARDS vs. DC | Endocrine and other factor-regulated calcium reabsorption | 1 | 0.02678 | 0.125 |
| ARDS vs. DC | Apelin signaling pathway | 1 | 0.030081 | 0.111111111 |
| ARDS vs. DC | Necroptosis | 1 | 0.033372 | 0.1 |
| ARDS vs. DC | Parathyroid hormone synthesis, secretion and action | 1 | 0.033372 | 0.1 |
| ARDS vs. DC | Calcium signaling pathway | 1 | 0.036652 | 0.090909091 |
| ARDS vs. DC | Phospholipase D signaling pathway | 1 | 0.036652 | 0.090909091 |
| ARDS vs. DC | Choline metabolism in cancer | 1 | 0.036652 | 0.090909091 |
| ARDS vs. HC | Choline metabolism in cancer | 2 | 0.002944 | 0.181818182 |
| ARDS vs. HC | Sphingolipid signaling pathway | 2 | 0.005516 | 0.133333333 |
| ARDS vs. HC | Lysine degradation | 3 | 0.008344 | 0.053571429 |
| ARDS vs. HC | Glycerophospholipid metabolism | 3 | 0.008344 | 0.053571429 |
| ARDS vs. HC | Sphingolipid metabolism | 2 | 0.017432 | 0.074074074 |
| ARDS vs. HC | Linoleic acid metabolism | 2 | 0.018686 | 0.071428571 |
| ARDS vs. HC | Mineral absorption | 2 | 0.019976 | 0.068965517 |
| ARDS vs. HC | Autophagy - other | 1 | 0.022675 | 0.333333333 |
| ARDS vs. HC | Apoptosis | 1 | 0.030123 | 0.25 |
| ARDS vs. HC | Bile secretion | 4 | 0.040632 | 0.022988506 |
| ARDS vs. HC | Autophagy - animal | 1 | 0.044855 | 0.166666667 |
| ARDS vs. HC | Fc gamma R-mediated phagocytosis | 1 | 0.059372 | 0.125 |
| ARDS vs. HC | Endocrine and other factor-regulated calcium reabsorption | 1 | 0.059372 | 0.125 |
| DC vs. HC | Choline metabolism in cancer | 2 | 0.002731 | 0.181818182 |
| DC vs. HC | Lysine degradation | 3 | 0.0075 | 0.053571429 |
| DC vs. HC | Glycerophospholipid metabolism | 3 | 0.0075 | 0.053571429 |
| DC vs. HC | Bile secretion | 5 | 0.007619 | 0.028735632 |
| DC vs. HC | Mineral absorption | 2 | 0.01859 | 0.068965517 |
| DC vs. HC | Autophagy - other | 1 | 0.021842 | 0.333333333 |
| DC vs. HC | Apoptosis | 1 | 0.02902 | 0.25 |
| DC vs. HC | Autophagy - animal | 1 | 0.043225 | 0.166666667 |

**Table S3. The DAP between deceased and survived ARDS.**

| Gene | logFC | AveExpr | P.Value | adj.P.Val | Compare |
| --- | --- | --- | --- | --- | --- |
| TBCB | -0.6719282 | -0.2291773 | 0.00011709 | 0.02100244 | deceased-survived |
| TMED2 | -0.6671753 | 0.0218195 | 0.00029617 | 0.03436854 | deceased-survived |
| TAGLN2 | -0.6439316 | -0.2399998 | 0.00028031 | 0.03436854 | deceased-survived |
| HSDL2 | -0.6211779 | -0.2693525 | 0.00025766 | 0.03436854 | deceased-survived |
| SYK | -0.6197709 | -0.3415852 | 0.00011693 | 0.02100244 | deceased-survived |
| EPX | -0.6071317 | -0.1342443 | 0.00068158 | 0.04664486 | deceased-survived |
| ASAP2 | -0.5891296 | -0.2500608 | 0.00076285 | 0.04837122 | deceased-survived |
| ACIN1 | 0.58575861 | 0.42698586 | 0.00021336 | 0.03436854 | deceased-survived |
| PPP1R7 | 0.58814633 | 0.34767383 | 0.00047191 | 0.0393603 | deceased-survived |
| PTMA | 0.6045931 | 0.39667824 | 0.00025496 | 0.03436854 | deceased-survived |
| HRNR | 0.61518606 | -0.0516892 | 0.00079743 | 0.04837122 | deceased-survived |
| TBCA | 0.61561094 | 0.18626459 | 0.00039052 | 0.03529722 | deceased-survived |
| NACA | 0.61658488 | 0.28188036 | 0.00034666 | 0.03516728 | deceased-survived |
| CAPN2 | 0.618448 | 0.14428018 | 0.00028681 | 0.03436854 | deceased-survived |
| FLG2 | 0.61912563 | -0.0521589 | 0.0008417 | 0.04992245 | deceased-survived |
| LBP | 0.62097518 | 0.11844295 | 0.00062092 | 0.0460344 | deceased-survived |
| SPON1 | 0.62418812 | 0.18608899 | 0.00070632 | 0.04712951 | deceased-survived |
| RANGAP1 | 0.6277671 | 0.08016607 | 0.00054207 | 0.04255228 | deceased-survived |
| WDR44 | 0.63040208 | 0.18540633 | 0.00060125 | 0.04584937 | deceased-survived |
| LDHB | 0.63210231 | 0.317993 | 0.00034889 | 0.03516728 | deceased-survived |
| MYL4 | 0.63240925 | 0.10408419 | 0.0006629 | 0.04655991 | deceased-survived |
| PDIA4 | 0.65622246 | 0.19475527 | 0.00039675 | 0.03529722 | deceased-survived |
| PSMA1 | 0.66251461 | 0.08951768 | 0.00025714 | 0.03436854 | deceased-survived |
| KRT1 | 0.66352305 | -0.0131546 | 0.00035576 | 0.03516728 | deceased-survived |
| CTSL | 0.67223585 | -0.0108752 | 0.0004274 | 0.03679789 | deceased-survived |
| HTRA1 | 0.67997647 | 0.07855264 | 0.00031773 | 0.03516728 | deceased-survived |
| EEF2 | 0.68456994 | 0.24878709 | 5.4273E-05 | 0.01448552 | deceased-survived |
| SPP1 | 0.68684432 | 0.24480926 | 8.1352E-05 | 0.01885677 | deceased-survived |
| KRT9 | 0.68903696 | 0.0070054 | 0.0002254 | 0.03436854 | deceased-survived |
| PTMS | 0.71103215 | 0.25342387 | 8.4781E-05 | 0.01885677 | deceased-survived |
| NUCB1 | 0.71190102 | 0.18607182 | 0.00011804 | 0.02100244 | deceased-survived |
| CAND1 | 0.74384901 | 0.20208132 | 3.9031E-05 | 0.011575 | deceased-survived |
| PSME1 | 0.77162955 | 0.17392586 | 7.7378E-06 | 0.00295032 | deceased-survived |
| EZR | 0.78369871 | 0.28873514 | 1.0193E-05 | 0.0034005 | deceased-survived |
| PSME2 | 0.78386074 | 0.09393115 | 6.9509E-06 | 0.00295032 | deceased-survived |
| CAPNS1 | 0.79289945 | 0.26926305 | 4.2208E-06 | 0.00225308 | deceased-survived |
| LMNA | 0.8143974 | 0.2250422 | 3.4629E-06 | 0.00225308 | deceased-survived |
| NUDC | 0.86362674 | 0.16892193 | 1.0683E-06 | 0.00126698 | deceased-survived |
| MSN | 0.88650524 | 0.23241071 | 1.4241E-06 | 0.00126698 | deceased-survived |
| RDX | 0.91389513 | 0.22085216 | 5.4519E-07 | 0.00126698 | deceased-survived |

**Reference:**

1. Tang SS, Wang YZ, Luo RK, Fang RD, Liu YF, Xiang H, Ran P, Tong YX, Sun MJ, Tan SB *et al*: **Proteomic characterization identifies clinically relevant subgroups of soft tissue sarcoma**. *Nat Commun* 2024, **15**(1).
